# Gene design, optimization of protein expression and preliminary evaluation of a new chimeric protein for the serological diagnosis of both human and canine visceral leishmaniasis

**DOI:** 10.1371/journal.pntd.0008488

**Published:** 2020-07-27

**Authors:** Wagner J. T. Santos, Diego H. C. Tavares, Artur L. Castro Neto, Marília B. Nascimento, Rafael Dhalia, Alessandra L. Albuquerque, Carlos H. N. Costa, Franklin B. Magalhães, Antônio M. Rezende, Osvaldo P. de Melo Neto

**Affiliations:** 1 Instituto Aggeu Magalhães (IAM)–Fundação Oswaldo Cruz (Fiocruz), Recife, Pernambuco, Brazil; 2 Universidade Federal de Pernambuco, Recife, Pernambuco, Brazil; 3 Universidade Federal do Piaui, Teresina, Piaui, Brazil; 4 Centro Universitário Tabosa de Almeida, Caruaru, Pernambuco, Brazil; Bernhard Nocht Institute for Tropical Medicine, Hamburg, Germany, GERMANY

## Abstract

**Background:**

Visceral leishmaniasis (VL) is a major neglected disease, potentially fatal, whose control is still impaired by inefficient and/or expensive treatment and diagnostic methods. The most promising approach for VL diagnosis uses serological assays with recombinant proteins, since they are more efficient and easier to perform. Tests developed for the human form of the disease, however, have not been shown to be efficient for its diagnosis in the canine host, the major reservoir for the American VL.

**Methodology/Principal findings:**

Here, we describe a systematic approach aimed at the production of a new chimeric protein potentially able to be used for both human and canine VL diagnosis and based both on *in silico* gene design and experimental data. Starting from the previous identification of *Leishmania infantum* recombinant antigens efficient for the diagnosis of either human or canine VL, three of the best performing antigens were selected (Lci2, Lci3 and Lci12). After a preliminary evaluation validating the chimeric approach, DNA fragments encoding predicted antigenic regions from each protein, enriched with repeats, were joined in various combinations to generate a total of seventeen chimeric genes optimized for prokaryotic expression. These were assessed for optimal expression and purification yield, with four chimeric proteins being efficiently produced. Their diagnostic potential was then evaluated through ELISA assays with sera from VL afflicted humans and dogs. After two rounds of gene design, the results showed high levels of sensitivity for the best chimeric protein, named Q5, in humans (82%) and dogs (100%) with 100% specificity in comparison with healthy controls. A single non-specific reaction was seen with serum from individuals with tegumentary leishmaniasis.

**Conclusion:**

The newly described chimeric protein is potentially useful for the detection of both humans and dogs afflicted with VL, with its use in rapid tests necessary for validation as a new diagnostic tool.

## Author summary

Visceral leishmaniasis (VL) is a major neglected disease, potentially fatal, caused mainly by *Leishmania infantum* and *L*. *donovani*. Its control is still impaired by inefficient and/or expensive treatment and diagnostic methods. VL diagnosis is mostly dependent on serological assays made with bacterially expressed *Leishmania* proteins. Tests developed for the human form of the disease, however, are not efficient for its diagnosis in the canine host. Dogs are the major reservoir for the American VL and their infection also needs to be monitored, requiring a distinct diagnostic test. Here, we describe a new chimeric protein potentially able to be used for both human and canine VL diagnosis. Based on a systematic approach using three *Leishmania* proteins known to be efficient for the diagnosis of either human or canine VL, fragments of each were joined in various combinations. The diagnostic potential of different chimeric proteins was then evaluated with human and canine sera from VL afflicted individuals. The best protein showed high levels of sensitivity in humans and dogs with no relevant false positive results with healthy controls or humans with tegumentary leishmaniasis. It is then potentially useful for the detection of both humans and dogs afflicted with VL in novel diagnostic tests.

## Introduction

The leishmaniases are a group of infectious-parasitic diseases caused by flagellated protozoa belonging to the family Trypanosomatidae, genus *Leishmania*. These parasites have a heteroxenic life cycle, living alternatively in vertebrate hosts (man and other wild and/or domestic mammals) and insect sandfly vectors. The leishmaniases are found in most tropical and subtropical countries and are considered a major global public health problem [1]. In humans, these diseases are present in three main clinical forms: cutaneous, mucocutaneous and visceral leishmaniasis. The visceral form of the disease, potentially fatal if not treated, is caused by *Leishmania donovani* (Old World) and *L*. *infantum* (New World and Old World) [2]. In many regions, the domestic dog has a major role for the visceral leishmaniasis (VL) epidemiology, not only due to its high infection prevalence, compared to humans, but also due to the large number of asymptomatic animals that host the parasite and are reservoirs for the disease [3–5].

There are currently many methods for the VL diagnosis, each having different applications and sensitivities. Several studies have shown that serological diagnosis is the most appropriate approach to be used in endemic regions, since it is easy to use, non-invasive and rapidly generates results. However, despite the many choices available to be used in the field, no test so far is efficient enough to be used to diagnose the disease in both humans and dogs [6–9]. Among the serological methods currently used (DAT, IFAT, ELISA and immunochromatographic tests) the ELISA is the most efficient, when using recombinant proteins [10]. ELISA based on the rK39 antigen is the most common test used for human VL diagnosis, with high sensitivity results (67–99%) [11,12]. Alternatively, the rK39 immunochromatographic (IC) test, despite being considered an efficient test for human VL, shows variable sensitivities when used in different regions of the world. Studies in India showed a 97% sensitivity and 90% specificity, but in East Africa the results with the same test showed 85% sensitivity/ 91% specificity [13]. Regarding canine VL diagnosis, the rK39 serological tests displayed lower sensitivities (less than 80%), in both ELISA and IC tests. Nevertheless, a chimeric protein based on rK39 and other antigens (named rK28) performed significantly better and is currently the basis for the commercially produced dual-path platform (DPP) test, [5,14,15].

Through the screening of genomic and cDNA expression libraries, we have previously identified several novel *L*. *infantum* antigens with the potential to improve the serological diagnosis of either dogs or humans afflicted with VL [16–18]. However, none of these antigens proved to be efficient for the diagnosis of both human and canine VL. Mixtures of proteins with the best results for the detection of human and dog VL were also tested and these performed better [16]. However, the production of a diagnostic system with two, three or more recombinant proteins, would increase costs and complicate standardization. An alternative is the use of chimeric polypeptides based on the previously characterized antigens. Here we report the generation of a new chimeric protein (Q5), based on three of the previously described antigens (Lci2, Lci3 and Lci12), that showed efficient sensitivity and specificity for diagnosing VL afflicted dogs as well as humans.

## Methods

### Optimization and synthesis of recombinant antigens and chimeric genes

Synthetic genes encoding the Lci2, Lci3 and Lci12 antigens were designed and optimized for heterologous expression in eukaryotic cells with the LETO 1.0 program, taking into consideration the reading phase, codon frequency, mRNA secondary structure, GC content and restriction sites. They were then manufactured by GENEART (Life Tech, St. Paulo, Brazil) cloned into the pMK plasmid. Using the added 5’ NheI site and the pMK encoded 3’ HindIII site, the synthesized sequences were then subcloned in frame into the NheI / HindIII sites of the pRSETa expression vector (Invitrogen). To generate the Lci2-R construct, the fragment encoding the protein’s N-terminal region was removed by XhoI digestion followed by plasmid + insert purification and religation. For Lci12-NR, the fragment encoding the repeats was removed after digestion with SalI and partial digestion with XhoI and also followed by plasmid + insert purification and religation.

To generate the first set of chimeric genes (D1, D2 and D3), a total of three successive subcloning steps were required for each gene (as exemplified for D1 in the Supporting S1 Fig). In the first subcloning, the XhoI (partial digestion) / EcoRI enzymes were used to excise the entire inserts from the pMK plasmid and subclone them into the same sites of pRSETa. For the second and third steps, DNA fragments with the regions encoding the repeats from the three genes were first recovered by digestion with XhoI / SalI. The second step consisted of subcloning the fragment with the Lci3 repeats into the XhoI site of the Lci2 containing plasmid, for the D1 construct, while the fragments with the Lci2 and Lci12 repeats were then subcloned into the same site of the Lci3 plasmid, respectively, for D2 and D3. In the correct orientation the ligation of these fragments eliminated the complementary XhoI / SalI 3’ sites, leaving a XhoI 5’ site. For the third and last step, the fragment with the Lci12 repeats was cloned into this XhoI site of the D1 and D2 constructs, while the Lci2 fragment was cloned into the equivalent site of D3. Again, a single 5’ end XhoI site remained for the correctly oriented constructs, which were then confirmed through sequencing.

### Generation of *in silico* designed chimeric genes

Prior to gene design, a prediction of linear B cell epitopes in the sequences of the selected protein regions was first performed using the BCPred1.2 program [19] to provide support that the selected regions of Lci2, Lci3, Lci12 and Lci13 were antigenic. Sequences of the genes optimized for expression in *Escherichia coli* were designed through the Gendesigner program [20,21] and sent for commercial synthesis to GenScript, (Piscataway, New Jersey, USA) for the first set of chimeric proteins (Q1 through Q4) and Thermo—GENEART (Life Tech, St. Paulo, Brazil), for Q5 only. The synthesized genes were purchased cloned into pUC57 and, upon arrival, subcloned into the XbaI / HindIII sites of the pRSETa vector (Thermo Life Tech, São Paulo, Brazil). This subcloning strategy removes the original plasmid encoded ribosomal binding site, translation initiation codon, poly-histidine tag and leader peptides, as well as most of the restriction sites used for cloning within the expression vector. After subcloning, different gene combinations were produced through digestion with different restriction enzymes, plasmid purification and religation, as described in the results section and exemplified in the Supporting S2 Fig (for Q1SX and Q1NN). All constructs were confirmed by restriction enzyme digestion and sequencing.

### Expression and purification of recombinant proteins

For protein expression, the various chimeric constructs were transformed into *E*. *coli* BL21 DE3 or Rosetta™ 2 DE3 (both from Novagen), followed by selection with ampicillin (50 μg/ml) and chloramphenicol (34 μg/ml) in 37ºC. Cells were cultured in liquid LB medium (500 ml) and the expression of recombinant proteins induced with 0.5 mM IPTG at 30ºC. Induced cell pellets were resuspended in 20 ml of denaturing lysis buffer (100 mM sodium phosphate, 10 mM Tris, 8 M Urea, 20 mM imidazole, pH 8.0) with most of the induced protein solubilized. Cell lysis was carried out by ultrasonication, in 5 pulses of 30 seconds with one minute intervals, at 4ºC. Protein purification was performed by incubation with Ni-NTA (400 μl) agarose beads (Qiagen, catalogue no. 30210), followed by washes in lysis buffer with 50 mM imidazole (pH 6.0) and elution in the same buffer with 1 M imidazole (pH 4.5), all under denaturing conditions. The purified proteins were quantified using the Bio-Rad Protein Assay Kit, based on the Bradford method, with an approximate yield of 1 mg of protein for each purification (for Q5 and related proteins). All protein preparations were visualized in 15% SDS-PAGE, stained with Comassie Blue R-250, and further quantified by comparisons with known quantities of BSA. For some of the purified proteins minor contaminants, of mostly lower molecular weight, could be seen, but this are likely degradation products which maintain the poly-histidine tags and do not seem to interfere with the assays carried out.

### Antibody production and Western blotting

Rabbit antisera were raised against Lci3 (originally named rLci3A-R3-NH6) and Lci12 by immunizing adult New Zealand White rabbits with the originally described His-tagged recombinant proteins [16,17]. The production and analysis of the Lci13 antiserum has been previously described [18]. To reduce the background, the various antibodies were affinity purified and stored in aliquots at -80°C prior to use. Western blotting was performed with the Immobilon-P polyvinylidene difluoride (PVDF) membrane (Millipore), using as secondary antibody the peroxidase-conjugated AffiniPure Goat Anti-Human IgG (Jackson ImmunoResearch Laboratories, catalogue no. 109-005-003). Alternatively, for the His-tag detection, a mouse monoclonal Anti-6X His tag (GE Healthcare, catalogue no. 27-4710-01) was used, with the AffiniPure Goat Anti-Mouse IgG monoclonal anti-mouse IgG (Jackson ImmunoResearch Laboratories, catalogue no. 115-005-003) as secondary antibody. The reactions were detected by enhanced chemiluminescence (ECL).

### Indirect ELISA assay

The ELISA was performed essentially as described previously [16,17]. For the standard assay, the recombinant proteins were diluted to 6 μg/ml in 50 mM Na_2_HCO_3_/NaHCO_3_ buffer (pH 9.6), with 100 μl added to each well of 96-well microtiter plates. This was followed by incubation at 4°C for 16 hours and blocking with Phosphate-Buffered Saline plus 0.05% Tween-20 (PBS-T), pH 7.2, supplemented with 10% dry non-fat milk. Wells were incubated with the selected sera at dilutions of 1:900 (human) or 1:200 (canine), for Lci2, Lci2-R, Lci12 and Lci12-NR, 1:6000 or 1:900 for D2 and 1:2500 or 1:900 for Q1SX, Q2SX, Q4SX and Q5, respectively. These dilutions were defined after they were seen to produce both fewer false negative and false positive results. After washing with PBS-T, the wells were incubated with peroxidase-conjugated goat anti-dog IgG (diluted 1:1.200, from Jackson ImmunoResearch Laboratories, catalogue no. 304-005-003) or anti-human IgG (diluted 1:10.000, the same used for the western-blots), and incubated for 1 hour at room temperature, followed by further washes with PBS-T. The enzymatic activity was revealed with 0.01% hydrogen peroxide and 0.01% orto-phenylenodiamine (OPD, from Sigma-Aldrich) in 0.1 M phosphate-citrate buffer, pH 5.0, and the plates read with the 490nm filter on a Benchmark Plus Microplate Manager 5.2 (BIO-RAD). For each serum/protein sample, assays were carried out in triplicates with the result coming from the average of the three samples subtracting the values for blank wells (with buffer only). Cut-off values for the ELISAs were defined as the average of the results obtained with the sera from healthy donors, plus three standard deviations.

### Human and canine sera and ethical considerations

All human sera were collected following approval of their use by the appropriate ethics committees. The VL positive group consisted of 50 sera from VL patients, with the clinical and laboratory examination confirmed by parasitological diagnosis, with their use approved by the Ethics Committee from the Federal University of Piaui (0116/2005). The control group consisted of 50 sera from healthy individuals living in a non-endemic VL area. These sera were included in a study approved by the Ethics Committee of the Brazilian Ministry of Health (25000.119007/2002-03). For the canine sera, these were obtained from 100 domestic or wandering dogs from a VL endemic area localized in the city of Jequié, state of Bahia, Brazil, with either parasitologically or PCR confirmed diagnosis, or both, with the sera used as negative control previously described [16,17]. The Ethical Commission on the Use of Animals from the Institute Gonçalo Moniz (FIOCRUZ) approved the use of the sera from the dogs in this study (Ceua, protocol number 040 / 2005). The tegumentary leishmaniasis sera were derived from a group of 50 infected human patients, with parasitologically confirmed diagnosis, plus 50 negative control sera, from the Pernambuco state and their use has been approved by the IAM/FIOCRUZ Ethics Committee, registered in CAEE 11083812.7.0000.5190.

### Statistical analysis of the ELISA results

Sensitivity, specificity, positive and negative predictive values and confidence interval parameters were estimated using the MedCalc program (version 12.3) (MedCalc Software, Ostend, Belgium). The dot plot was generated with the GraphPad Prism program (GraphPad Prism version 6.00 for Windows, GraphPad Software, La Jolla California USA).

## Results

### Evaluation of synthetic genes encoding selected antigens

The three proteins chosen for this study (Lci2, Lci3 and Lci12) have no sequence homology to each other, but all consist of multiple copies of small repeated motifs or repeats flanked by N- and C-terminal regions. Lci2 consists of repeats of 39 amino acid residues and was the best antigen for the diagnosis of human VL, among the thirteen recombinant proteins tested by us. Lci3 and Lci12, respectively based on repeats fourteen and eight residues long, were efficient for canine VL [16,17]. The three native proteins, however, are long and based on a very large number of repeats, so that efficient expression of the corresponding full-length polypeptides in heterologous systems is not viable. Here, to enhance their potential for expression in different systems, without loss of antigenicity, a synthetic gene was first designed *in silico* for each protein.

**Fig 1:**
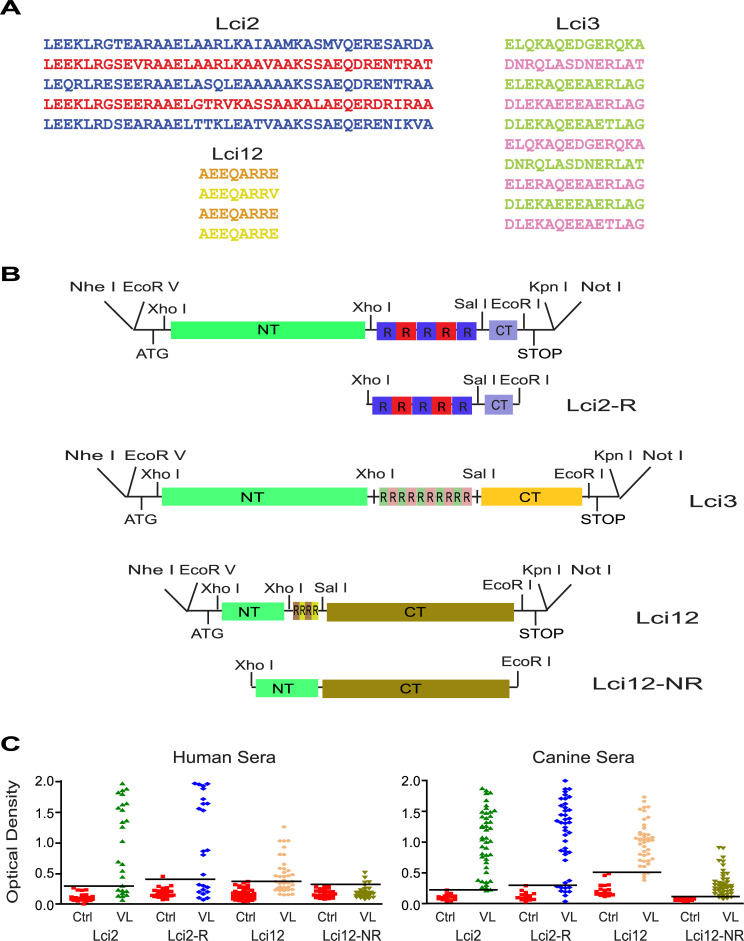
Schematic representation of the genes optimized for the expression of the truncated Lci2, Lci3 and Lci12 proteins and evaluation with human and canine sera. (A) Sequence of the repeats chosen to be included in the proteins encoded by the three synthetic genes. (B) Scheme detailing the organization of the optimized genes coding for the truncated variants of the three proteins selected for this study. The various regions with and without repeats are indicated, as well as relevant sites for the restriction enzymes used in cloning/subcloning procedures. (C) ELISA showing the evaluation of the different recombinant proteins described in the text with human and canine sera from VL afflicted individuals (VL) and healthy controls (Ctrl). NT (N–Terminal region) and CT (C–Terminal region).

The three synthetic genes consisted of both N- and C-terminal regions flanking a small set of repeats (five for Lci2, ten for Lci3 and four for Lci12), with the selected repeats being those most variable within the ones originally found. For each synthetic gene the number of encoded repeats was then defined based on the variations observed along their sequence within the original recombinant proteins (Fig 1A). Four repeats were chosen for Lci12 because all eight amino acid repeats are essentially identical with minimal variation. For Lci2, the 39 amino acid repeats are more diverse, but the repeats are larger, so five were chosen to maximize diversity without substantially increasing the final protein size. Also, for Lci3, the repeats are more diverse but considering their smaller size, 14 amino acids, more repeats were included, totaling 10. A schematic visualization of the synthetic genes generated for each of these three proteins can be seen in Fig 1B, with their full-length sequences shown in the Supporting S3–S8 Figs. Within the coding sequences, restriction enzyme sites were included to facilitate the exchange of genetic fragments. Two further constructs were also generated, in order to evaluate the contribution of the repeats to the proteins’ antigenicity (also shown in Fig 1B). The first one was derived from Lci2, after removal of the segment encoding the protein’s N-terminal region, and mostly consisted of the synthetic region with its repeats (Lci2-R); the second, derived from Lci12 after removal of the segment encoding its repeats, led to a Lci12 variant lacking repeats (Lci12-NR).

Expression in *Escherichia coli* and affinity purification of the corresponding recombinant proteins using a vector encoded, N-terminal, poly-histidine tag, was then attempted for all the different synthetic constructs. Only three proteins were successfully produced and found to be represented by single bands in SDS-PAGE gels: Lci2, Lci2-R and Lci12-NR. We first evaluated Lci2 and its Lci2-R derivative, tested through ELISA with sera from dogs and humans with VL, as well as with control sera from healthy individuals, as shown in Fig 1C. The two proteins were very sensitive for the diagnosis of both human and canine sera, confirming that the proteins encoded by the synthetic constructs display sensitivities equivalent to those observed for the protein derived from the native gene. Nevertheless, both had better performances with the canine samples, a result that is contrary to what was observed with the native protein, more efficient for human VL diagnosis [17].

For both canine and human sera, the differences in sensitivity observed between the full-length Lci2 and the truncated Lci2-R were small, with the segment having only the repeats being able to identify most positive sera with similar OD values. This is consistent with the repeats within the recombinant proteins having a major impact on the protein’s potential to be recognized by the positive sera. To evaluate this, we compared Lci12-NR with the previously described Lci12 recombinant protein [16]. With the canine sera, no significant differences in sensitivity results were seen between the two proteins, although Lci12-NR was associated with lower OD values (also shown in Fig 1B). In contrast, a large variation in sensitivity between them was observed for the human sera, with Lci12-NR having a much-reduced sensitivity in comparison to the original Lci12 protein. Again, an important contribution by the repeats is observed, which nevertheless may depend on the protein and sera set evaluated.

### Evaluation of a first set of chimeric proteins based on the Lci2, Lci3 and Lci12 antigens

**Fig 2:**
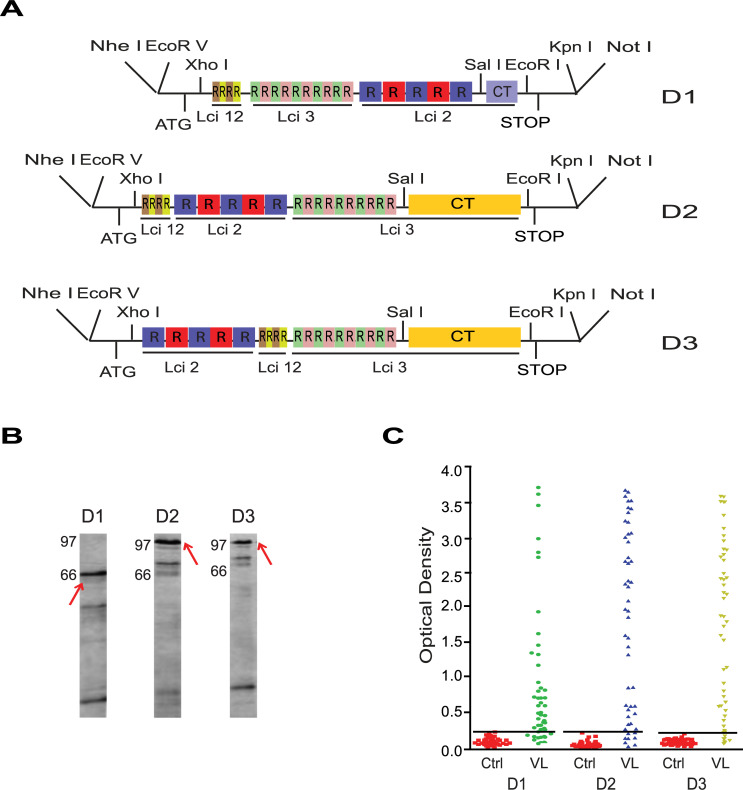
Preliminary assessment of a first set of chimeric proteins based on the Lci2, Lci3 and Lci12 antigens. (A) Schematic representation of the three chimeric proteins generated through subcloning of the Lci2, Lci3 and Lci12 synthetic gene fragments, as described in the text. The chimeric constructs differ in the order in which the different gene fragments are placed along the protein synthesis as well as in their C-terminal end (Lci2 for D1; Lci3 for D2 and D3) (the full-length DNA and protein sequences of the D2 chimera are shown in the Supporting S9 and S10 Figs). (B) SDS-PAGE gel evaluation of the corresponding chimeric proteins after expression in *Escherichia coli* and affinity purification. Numbers on the left indicate the sizes of molecular weight markers, with the red arrows highlighting the purified recombinant proteins. (C) ELISA comparing the recognition of the three chimeric proteins by human sera from VL afflicted and control individuals. CT (C-Terminal region).

To improve the diagnostic potential of the recombinant proteins for the diagnosis of both human and canine VL and to validate a chimeric protein approach, all three genes originally synthesized were subjected to multiple subcloning steps aiming to join the smaller gene fragments encoding the repeats from the different protein coding sequences. Three chimeric constructs were thus generated, named D1, D2 and D3, the first encoding the C-terminal region of Lci2 (D1) and the other two the C-terminus of Lci3 (D2 and D3). All three constructs encoded the full set of repeats from the three synthetic genes (Fig 2A), although their order varied from one chimeric construct to the order: D1 having the order Lci12, Lci3 and Lci2; D2 having Lci12, Lci2 and Lci3; and D3 with Lci2, Lci12 and Lci3. For the gene order we purportedly omitted using the C-terminal end of Lci12, substantially larger in size, with the order of the remaining fragments defined arbitrarily according to the success of the cloning strategies used. The three chimeric genes were all expressed in *Escherichia coli*, generating predicted bands that were affinity purified (Fig 2B) and then assayed for their ability to diagnose VL through ELISA with human sera. All three proteins were very efficient for VL diagnosis (Fig 2C) with the D2 construct marginally better than the others, having sensitivity values of 94%, in comparison with 89 and 87% for D1 and D3, respectively (summarized in Table 1). Remarkably, these positive values were achieved using sera diluted fivefold more than the dilutions used for the original, non-chimeric, recombinant proteins. The chimeric D2 was also tested with canine sera and again performed well, although not as efficient as with the human sera, with 76% sensitivity (also in Table 1). These results indicated that the chimeric proteins based on the previously described antigens are feasible alternatives for the improvement of serological diagnostic tests for human and canine VL. They also indicated that the order in which the antigenic fragments are placed in the chimeric proteins can influence their recognition by the tested sera, something that can only be evaluated experimentally. The synthetic genes, however, were not optimized for expression in *Escherichia coli* and the chimeric proteins proved to be difficult to produce and likely toxic, which impaired large scale validation assays.

**Table 1:** Sensitivity, specificity and accuracy values for the different chimeric proteins evaluated in this study with both sets of human and canine sera.

Proteins	Human sera (CI 95%)			Canine sera (CI 95%)		
	Sensitivity (N = 50)	Specificity (N = 50)	Accuracy	Sensitivity (N = 39)	Specificity (N = 15)	Accuracy
D1	89%	88%	92%			
D2	94%	100%	94%	76%	100%	82%
D3	87%	97%	89%			
Q1SX	72%	100%	86%	99%	100%	100%
Q2SX	76%	100%	83%	89%	100%	92%
Q4SX	32%	100%	58%	71%	100%	78%
Q5	82%	100%	92%	100%	100%	98%

### *In silico* designed chimeric genes

**Fig 3:**
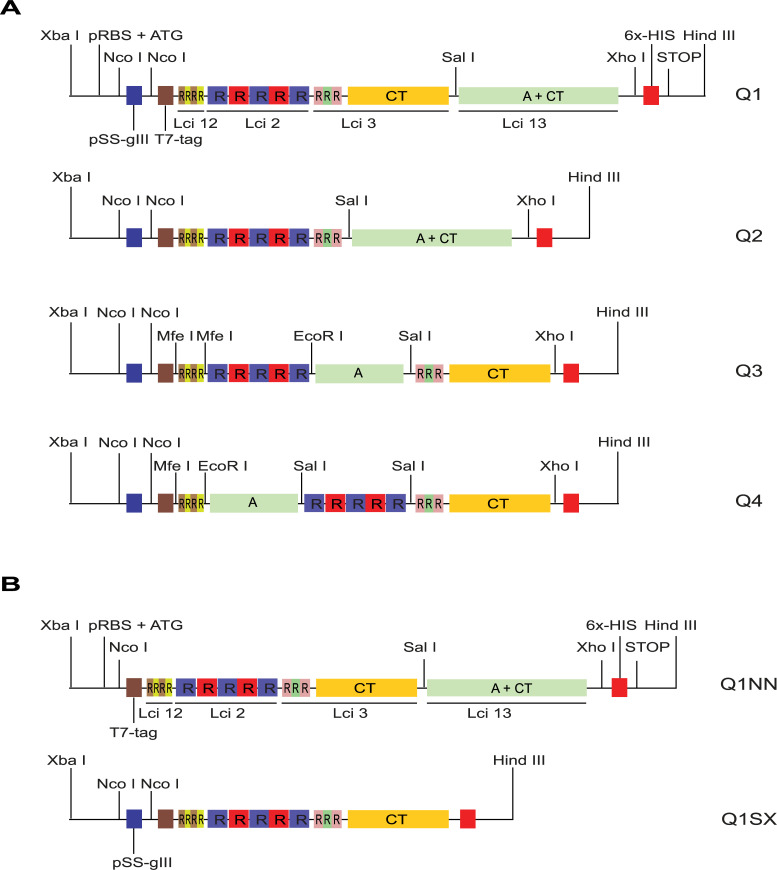
Schematic representation of the *in silico* designed chimeric genes evaluated here. (A) Representation of the four *in silico* designed chimeric genes originally synthesized (Q1 through Q4), indicating the fragments derived from the selected antigens as well as relevant restriction sites for further cloning procedures. (B) Representation of the constructs derived from the Q1 gene after removal of selected fragments through digestion with restriction enzymes and religation. Q1NN: Q1 after removal of the pSS-gIII tag through digestion with NcoI and religation; Q1SX: Q1 after removal of the Lci13-CT fragment, through digestion with SalI/XhoI and religation. CT (C-Terminal region), A (Antigenic region).

In order to generate chimeric proteins that would be effective for VL diagnosis as well as efficiently expressed in bacterial systems, we opted to go through a new round of chimeric gene design and synthesis. Aiming to improve the results with the canine sera, this time we opted to reduce to three the number of repeats for Lci3 while including a fourth antigen, Lci13, which was previously seen to have the best performance for canine VL diagnosis [16]. We also minimized cloning procedures and designed the chimeric constructs already having fragments of the different antigens in alternative positions, in all synthesizing four different chimeric genes, named Q1 through Q4 (Fig 3A). The four genes had their codons optimized for expression in *E*. *coli* but, in order to maximize expression, they also had included on their 5’ ends additional sequences to enhance expression. These include a ribosome binding site and Shine-Dalgarno element (pRBS), to stimulate mRNA translation, and sequences encoding two distinct N-terminal motifs from bacteriophage proteins to optimize protein expression and stabilization: the N-terminal leader motif from the protein III of filamentous bacteriophages (the pSS-gIII peptide), originally used for the efficient expression of protein fragments in phage-display libraries [22]; and the first eleven residues of the major capsid protein from the T7 bacteriophage (the T7 tag epitope–[23]). A six histidine (6x-His) encoding sequence was also included within the 3’ end of the genes, prior to the translation termination codon, to allow purification of the different proteins, independently of the expression vector used. Restriction enzyme sites were added at strategic positions along the sequences enabling further manipulation of these genes, by facilitating the exclusion of selected sequences, as described below.

Restriction enzyme digestions and religations were performed to generate truncated variants of the four original synthetic genes (Q1 through-Q4). This aimed to facilitate testing alternatives and possible antigenic combinations in order to improve the protein expression in the prokaryotic system. So, for all four genes, an alternative construct was generated by digestion with NcoI and religation after removal of the 75 bp fragment encoding the N-terminal peptide pSS-gIII (exemplified in Fig 3B for the Q1 gene), generating the NN variants, such as Q1NN. Using equivalent strategies, selected antigenic encoding fragments were also removed. For the Q1, for instance, the DNA fragment encoding the Lci13 C-terminal region was removed after digestion with SalI/XhoI and religation, generating the Q1SX construct (Fig 3B). Similar variants were also generated for Q2, Q3 and Q4, resulting in sixteen different chimeric constructs (listed on S1 Table).

### Expression profiles in *E*. *coli* of *in silico* chimeric proteins

**Fig 4:**
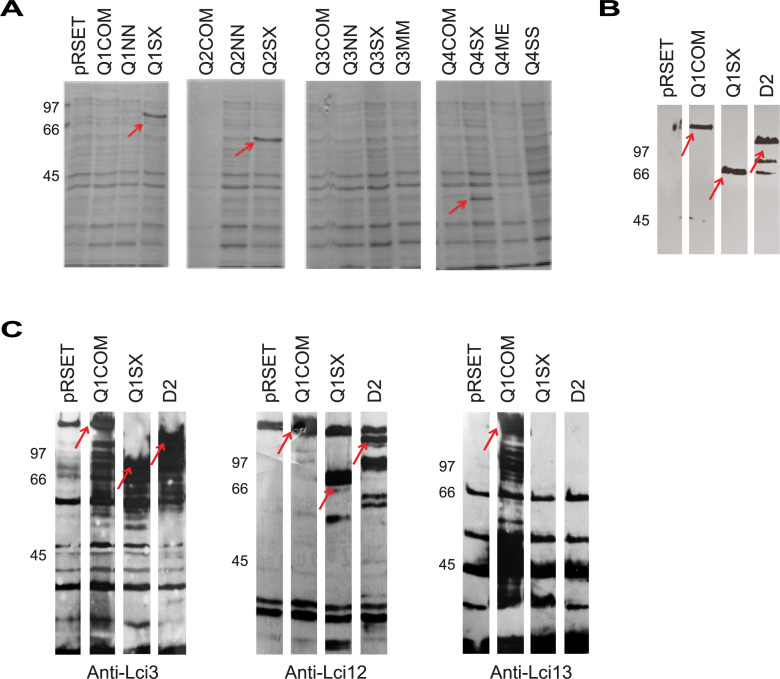
Expression of the *in silico* designed chimeric proteins in *E*. *coli*. (A) Representative Coomassie-Blue stained SDS-PAGE gel evaluating the expression of fourteen of the proteins encoded by the *in silico* designed chimeric genes. These were assessed in whole extracts of IPTG induced Rosetta 2 *Escherichia coli* cells. (B) Western blot assay assaying the recognition of selected chimeric proteins in whole *E*. *coli* extracts with a commercial anti-histidine monoclonal antibody. (C) Western blot evaluating the recognition of selected chimeric proteins with poly-clonal sera raised against the Lci3, Lci12 and Lci13 recombinant proteins. The red arrows highlight the bands corresponding to the expected sizes for the different chimeric proteins.

To produce the different recombinant proteins, all constructs derived from the synthetic chimeric genes were transformed into *E*. *coli* cells and their expression evaluated through SDS-PAGE gels. Recombinant protein expression was first evaluated after Coomassie Blue staining of whole cell extracts in two different *E*. *coli* cell lines (BL21 or Rosetta™ 2). Of all constructs evaluated, best results were seen for the Rosetta™ 2 cells, but even then, clearly identifiable, inducible, bands were only observed for the Q1SX, Q2SX and Q4SX constructs (Fig 4A). No differences between the full-length original proteins and corresponding NN variants were seen, indicating a lack of enhancement in expression mediated by the N-terminal pSS-gIII sequence. To confirm the expression of selected constructs, even in BL21 cells, and to confirm the presence of their C-terminal poly-histidine tag, bacterial extracts were then subjected to a Western Blot with an anti-His antibody (Fig 4B). These confirmed the expression of most proteins, despite the fact that many of these were not visible in the Coomassie Blue stained gels.

We next assayed the same extracts with polyclonal antisera directed against the Lci3, Lci12 and Lci13 antigens. In most cases these sera recognized the chimeric proteins having the corresponding protein sizes. For instance, all three sera recognized the Q1 full length, but only the Lci3 and Lci12 antisera recognized Q1SX, missing the Lci13 fragment (Fig 4C). Overall, our results show a very poor expression of the Q3 chimera and its derivatives in *E*. *coli*, indicating that the order in which the individual fragments were positioned was detrimental for protein expression. For the other three chimeric constructs, these results also highlight the greater expression efficiency for the lower molecular weight proteins, the SX variants.

### Assessment of selected chimeric proteins for the VL diagnosis

**Fig 5:**
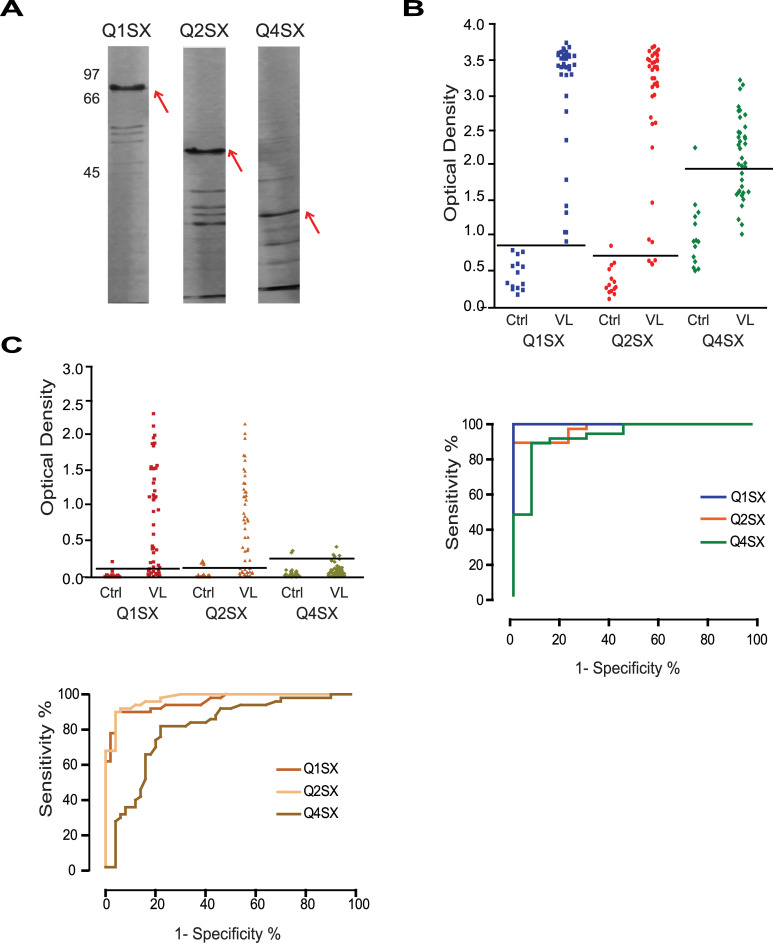
Serological evaluation of selected chimeric proteins. (A) SDS-PAGE gel showing the affinity purification of the proteins with best performances in expression in *Escherichia coli*. (B) and (C) Summary of the ELISA results and ROC curve analyses (top and bottom panels, respectively) of the chimeric proteins with sera from dogs and humans with VL, as well as healthy control individuals.

The three proteins having the best expression results (Q1SX, Q2SX and Q4SX) were then selected for large scale expression and affinity purification. In contrast to the recombinant proteins from the first round of chimeric gene design, all three expressed well in the large-scale settings and both Q1SX and Q2SX also were easily purified, although Q4SX did not purify well (Fig 5A). All three, nevertheless, were submitted to the evaluation of their diagnostic potential through ELISA with sera from dogs and humans with VL. With the canine sera, both Q1SX and Q2SX proteins performed very well, with a clear distinction between positive and negative samples for Q1SX and a slightly weaker performance for Q2SX (Fig 5B), with sensitivity values of 99 and 89%, respectively (Table 1). In contrast, a significant number of false negative samples were seen for the Q4SX protein, with an overall poorer performance and only 71% sensitivity. Against the human sera, all three proteins performed less efficiently, with all having many false negative samples and Q4SX also being much less efficient for VL diagnosis that than the other two proteins evaluated (Fig 5C). The sensitivity values for the Q1SX and Q2SX were nevertheless still satisfactory, respectively 72 and 76% (Table 1), although not ideal for diagnostic purposes. Results for Q4SX, however, the only protein having a fragment of the Lci13 antigen and missing the Lci3 segment, were insufficient, with only 32% of positive results.

### Design, synthesis and evaluation of a new chimeric gene and recombinant protein

**Fig 6:**
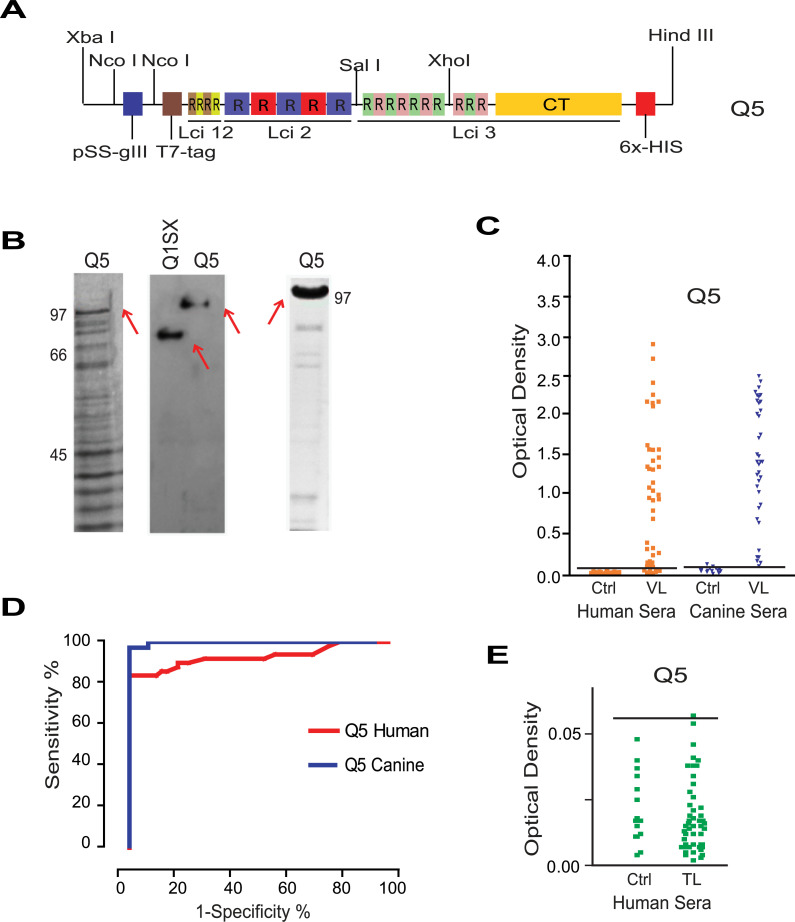
Design and evaluation of the new chimeric Q5 protein. (A) Schematic representation of the new Q5 construct designed with the extra Lci3 repeats (in comparison with Q1SX) flanked by Sal I/Xho I restriction sites. (B) SDS-PAGE and Western-blot evaluation of the Q5 recombinant protein. The left panel shows the Coomassie-Blue stained SDS-PAGE gel evaluation of the Q5 expression in whole cell bacterial extracts. The middle panel shows a western blot comparing the recognition of Q1SX and Q5 in whole bacterial extracts by an anti-his antibody, with the right panel confirming the affinity purification of the Q5 protein in another Coomassie-Blue stained gel. (C) ELISA assay evaluating the Q5 protein with both human and canine sera from VL afflicted individuals. (D) ROC curve analyses of the Q5 protein proteins with sera from dogs and humans with VL. (E) Same as (C) but comparing the recombinant Q5 with human sera from individuals with tegumentary leishmaniasis (TL) as well as corresponding negative controls.

Considering the greater sensitivity for the human sera achieved with the chimeric protein from the first round of gene design (D2–94% sensitivity), much better than a very similar protein from the second round (Q1SX– 72% sensitivity), we considered the differences between the two proteins. Although nearly identical, the D2 protein included seven extra repeats from Lci3 that were missing from Q1SX (DNA and protein sequences shown in the Supporting S11 and S12 Figs, with an alignment comparing the D2 and Q1SX proteins shown in S13 Fig). To increase sensitivity to human sera, while maintaining sensitivity to canine sera, these repeats were reinserted *in silico* in the Q1SX sequence and a new chimeric gene synthesized, named Q5 (DNA and protein sequences shown in the Supporting S14 and S15 Figs). The difference between the Q1SX and Q5 chimeras, then, was the fragment encoding the extra Lci3 repeats, flanked by Sall and XhoI sites, shown in the scheme from Fig 6A. After synthesis, subcloning and expression, the novel chimera was efficiently expressed and demonstrated to be recognized by the anti-poly-histidine marker antibody. Efficient expression was maintained by large-scale induction in *E*. *coli* and the protein was also efficiently affinity purified (Fig 6B).

The Q5 protein was next evaluated with human sera from individuals proven to be infected with *L*. *infantum*, sera from healthy controls, sera from dogs carrying VL and the healthy controls (Fig 6C and Fig 6D). The Q5 ELISA results showed an 82% sensitivity for human sera, while the results with sera from dogs maintained a sensitivity of 100%, with a 95% confidence interval (Table 1). Based on the improved results obtained for Q5, it was used in a new ELISA assays to evaluate its cross-reactivity with sera from patients with tegumentary leishmaniasis (Fig 6E). The results with these sera showed a single non-specific reaction among the 50 sera tested (2%), confirming the Q5 specificity for the VL diagnosis and its potential for minor cross-reactions with other diseases.

## Discussion

So far, there is still no diagnostic test available with a high efficacy for both canine and human VL, a tool which would facilitate acquisition and distribution in low income areas and would accelerate the monitoring of the disease in the canine host. Its development is impaired by the markedly distinct diagnostic performances observed for the same recombinant proteins with either canine or dog sera, as more recently noticed by us and others [16,17,24]. Joining segments encoding antigenic fragments from multiple proteins increase the diagnostic potential of the final chimeric polypeptides, but this can enhance or create expression problems. Larger molecules, despite having genes optimized for bacterial expression, are also more susceptible to enhanced misfolding or proteolysis events and these might justify the better results we have generally seen with the smaller polypeptides. Overall, the stepwise approach described here, starting with genes encoding proteins efficiently recognized by either the human or canine sera and improving them using bioinformatic tools and multiple rounds of protein expression and serological evaluations, might result in a relevant new tool for the diagnosis of both human and canine VL. Our results also highlight the importance that proteins having multiple repeats might have in inducing a greater serological response that can be used for diagnostic purposes.

Early on, many studies identified and evaluated chimeric proteins to be used as constituents of diagnostic tools and these were indeed shown to be responsible for increasing the sensitivity of diagnostic tests for a number of infectious diseases. These include tests for malaria [25], toxoplasmosis [26], tuberculosis [27], Chagas’ disease [28] and dengue [29]. With *Leishmania*, a relevant pioneering study using a chimeric antigen for the diagnosis of canine VL showed that joining epitopes from different proteins could enhance its diagnostic potential [30]. In a subsequent study with a single chimeric polypeptide, joining fragments derived from the rK39 antigen with those from two related proteins, named K9 and K26, very high levels of sensitivity and specificity were observed for both canine and human sera, with a significant improved performance for the canine sera when compared with an individual assessment of the best regions of the original proteins [31]. This chimeric polypeptide, the rK28 antigen, was also evaluated on a rapid test platform using human sera but, although at first it showed an improvement when compared with rK39 [32], follow up studies found equivalent performances for both rK39 and rK28 based tests, at least in some VL endemic countries [33]. As for canine VL diagnosis, rK28 is the basis for the commercially used DPP test in Brazil, however its performance has been proposed to be more compatible with its use as a screening or confirmatory test [5,34].

For the chimeric proteins described here, Lci2 was chosen to enhance the human diagnosis, due to its previous performance and its relatedness to the commercially used rK39 antigen. Both Lci2 and rK39 consist mainly of repeats of a 39 amino acids motif and are derived from the same *L*. *infantum*/*L*. *donovani* protein, a N-terminal kinesin belonging to the kinesin-3 family and that localizes to the *Leishmania* cortical cytoskeleton [35]. However, while rK39 consists of part of the protein’s N-terminal segment plus a subsequent set of 6.5 repeats [36], Lci2 is derived from its much shorter C-terminal end plus the neighbouring repeats [17]. The Lci2 repeats are generally more diverse in sequence, with this diversity being incorporated in the chimeric proteins evaluated here and likely accounting for a better performance.

All three proteins that are part of the chimeric antigens tested here (Lci2, Lci3 and Lci12) are rich in repeats, as also observed for the three proteins constituents of rK28, despite having the markedly contrasting performances with the human and canine VL sera. The potential use of proteins having multiple tandem repeats for diagnosis purposes in *Leishmania* has been highlighted before [37–39] and here it has been reinforced not only with the first set of recombinant proteins evaluated, but also when comparing the Q1 and Q5 proteins, differing in the number of repeats for the Lci3 antigen. Noteworthy, some of the extra Lci3 repeats included within Q5 also add more diversity in sequence and this also might have been responsible for its enhanced performance. Remarkably, both K9 and K26 components or rK28 are characterized by 14 amino acid repeats enriched in positively/negatively charged residues [40], as seen for Lci3, despite major differences in sequence. For the Q1/Q5 proteins, however, the inclusion of the Lci12 repeats adds a component of extra diversity not seen elsewhere.

Overall, the performance achieved here using the best chimeric proteins, mainly Q5, justifies further validation in diagnostic tests, both using the ELISA assay as well as a rapid test platform. If necessary, these proteins can also be further modified, in a rapid manner, enhancing their potential for a more widespread use in VL monitoring. The availability of a single, low-cost and rapid test for both human and canine forms of VL would greatly facilitate its acquisition and distribution to low-income areas and might therefore significantly enhance the strategies available to control this neglected disease.

## Supporting information

S1 Table Full set of gene constructs generated and evaluated in this manuscript. (PDF)

S1 Fig Schematic representation of the subcloning strategies used to generate the first set of chimeric proteins evaluated here (D1, D2 and D3). The figure illustrates the strategy used to generate D1, but identical strategies were used for the other two chimeric constructs. In all, the DNA fragments encoding the repeats were flanked by XhoI and SalI restriction sites and could be recovered by digestion with these enzymes, purified and ligated into the XhoI site of the recipient construct, with the correct orientation defined by restriction enzyme digestion/sequencing. Since SalI and XhoI have complementary overhang sequences, ligation could be achieved, but both sites were eliminated when ligated. A single 5’ XhoI site remained then after the subcloning, at the fragment’s 5’ end, when in the correct orientation. This site could then be used in a subsequent subcloning step.
(PDF)

S2 Fig Schematic representation of the strategies used to modify the second set of chimeric proteins (Q1, Q2, Q3 and Q4). The figure illustrates the strategy used to generate Q1SX and Q1NN constructs from Q1, but identical strategies were used for the other constructs. In all, removal of selected DNA fragments was carried out by restriction enzyme digestions (SalI and XhoI for Q1SX or NcoI for Q1NN), followed by purification of the larger, plasmid plus Q1, fragment and religation. The full set of restriction enzymes used are listed in S1 Table.
(PDF)

S3 Fig Full length nucleotide sequence of the synthetic Lci2 gene after cloning within the pRSET vector. The sequence also shows the segment encoding the N-terminal His-Tag from the vector (in red). The Nhe I, Sal I, EcoR I and Not I restriction sites are underlined, while the two Xho I sites are underlined and in italic. The TGA stop codon is in pink.
(PDF)

S4 Fig Full length amino acid sequence of the synthetic Lci2 recombinant protein after cloning within the pRSET vector. The sequence also shows the N-terminal His-Tag encoded by the vector (in red) and elements introduced during the synthesis and cloning procedures in purple. The N-terminus, the region encoding the repeats and the C-terminal segments are in green, orange and blue, respectively.
(PDF)

S5 Fig Full length nucleotide sequence of the synthetic Lci3 gene after cloning within the pRSET vector. The sequence also shows the segment encoding the N-terminal His-Tag from the vector (in red). The Nhe I, Sal I, EcoR I and Not I restriction sites are underlined, while the two Xho I sites are underlined and in italic. The TGA stop codon is in pink.
(PDF)

S6 Fig Full length amino acid sequence of the synthetic Lci3 recombinant protein after cloning within the pRSET vector. The sequence also shows the N-terminal His-Tag encoded by the vector (in red) and elements introduced during the synthesis and cloning procedures in purple. The N-terminus, the region encoding the repeats and the C-terminal segments are in green, orange and blue, respectively.
(PDF)

S7 Fig Full length nucleotide sequence of the synthetic Lci12 gene after cloning within the pRSET vector. The sequence also shows the segment encoding the N-terminal His-Tag from the vector (in red). The Nhe I, Sal I, EcoR I and Not I restriction sites are underlined, while the two Xho I sites are underlined and in italic. The TGA stop codon is in pink.
(PDF)

S8 Fig Full length amino acid sequence of the synthetic Lci12 recombinant protein after cloning within the pRSET vector. The sequence also shows the N-terminal His-Tag encoded by the vector (in red) and elements introduced during the synthesis and cloning procedures in purple. The N-terminus, the region encoding the repeats and the C-terminal segment are in green, orange and blue, respectively.
(PDF)

S9 Fig Full length nucleotide sequence of the recombinant D2 gene within the pRSET vector. The sequence also shows (in black) the segments introduced by the vector, with the region encoding the his-tag in red and elements introduced by the cloning procedures in purple and the stop codon in pink. The Xho I and Eco RI flanking sites are underlined. Fragments encoding the repeats from Lci12, Lci2 and Lci3 are in green, orange and blue, respectively. The Lci3 fragment lacking repeats is in brown.
(PDF)

S10 Fig Full length amino acid sequence of the recombinant D2 protein within the pRSET vector. The sequence also shows the N and C-terminal segments encoded by the vector (in black) with the histidine tag highlighted (in red) and elements introduced during the synthesis and cloning procedures in purple. The fragments encoding the repeats from Lci12, Lci2 and Lci3 are in green, orange and blue, respectively. The Lci3 fragment lacking repeats is in brown.
(PDF)

S11 Fig Full length nucleotide sequence of the recombinant Q1SX gene. The sequence also shows the flanking XbaI and HindIII sites, underlined. The segment encoding the pSS-gIII peptide, flanked by the two NcoI sites, is in pink, while the segment encoding the T7 tag epitope is in light blue. Segments encoding the repeats from Lci12, Lci2 and Lci3 are in green, orange and dark blue, respectively, while the fragment encoding the His-Tag is in red.
(PDF)

S12 Fig Full length amino acid sequence of the recombinant Q1SX protein. The sequence also shows the pSS-gIII peptide, in pink, and the T7 tag epitope, in light blue. Fragments corresponding to the regions encoding the repeats from Lci12, Lci2 and Lci3 are in green, orange and dark blue, respectively, and elements introduced during the synthesis and cloning procedures in purple. The C-terminal His-Tag is in red. The Lci3 fragment lacking repeats is in brown.
(PDF)

S13 Fig Alignment comparing the amino acid sequences of the D2 and Q1SX chimeras, highlighting the Lci3 repeats missing from Q1SX. The repeats found in D2, but missing from Q1SX, are boxed
(PDF)

S14 Fig Full length nucleotide sequence of the recombinant Q5 gene. The sequence also shows the flanking XbaI and HindIII sites, as well as the internal SalI and XhoI sites flanking the segment encoding the extra Lci3 repeats, in gray (all restriction sites are underlined). The segment encoding the pSS-gIII peptide, flanked by the two NcoI sites, is in pink, while the segment encoding the T7 tag epitope is in light blue. Segments encoding the repeats from Lci12, Lci2 and Lci3 also found in the Q1 constructs are in green, orange and dark blue, respectively, while the fragment encoding the His-Tag is in red.
(PDF)

S15 Fig Full length amino acid sequence of the recombinant Q5 protein. The sequence also shows the pSS-gIII peptide, in pink, and the T7 tag epitope, in light blue. Fragments corresponding to the regions encoding the repeats from Lci12 and Lci2 are in green, orange, respectively. The original Lci3 repeats segment found in the Q1 constructs is in dark blue, while the extra Lci3 repeats segment is in grey and its non-repeats region in brown. Elements introduced during the synthesis and cloning procedures are in purple. The C-terminal His-Tag is in red.
(PDF)
