# Supplementary material for: Gene design, optimization of protein expression and preliminary evaluation of a new chimeric protein for the serological diagnosis of both human and canine visceral leishmaniasis
Source: PLoS Negl Trop Dis. 2020 Jul 27;14(7):e0008488. doi: 10.1371/journal.pntd.0008488 (PMC7410341; doi:10.1371/journal.pntd.0008488)
Supplement: S1 Table — (PDF) [file pntd.0008488.s001.pdf]

Supporting Table S1. Full set of gene constructs generated and evaluated in this manuscript.

Nomenclature	Restriction sites used for the final construct	Size in bp of the final gene product	Predicted molecular weight of the protein (in kDa)	Expression in *E. coli* Coomassie*
Q1	-	2845	102.65	-
Q1NN	NcoI	2734	99.26	-
Q1SX	SalI/XhoI	1690	60.46	++
Q2	-	2430	87.07	-
Q2NN	NcoI	2319	83.68	-
Q2SX	SalI/XhoI	1275	44.88	++
Q3	-	2360	84.76	+
Q3NN	NcoI	2249	81.37	-
Q3SX	SalI/XhoI	1517	53.56	-
Q3MM	MfeI	2205	80.48	-
Q3ME	MfeI/EcoRI	1719	63.21	-
Q4	-	2358	84.35	-
Q4NN	NcoI	2247	81.21	-
Q4SX	SalI/XhoI	923	32.03	++
Q4ME	MfeI/EcoRI	2202	80.18	-
Q4SS	SalI	1716	63.28	-

* Expression in Rosetta2, as seen in Coomassie Blue stained gels.
