# Supplementary material for: Gene design, optimization of protein expression and preliminary evaluation of a new chimeric protein for the serological diagnosis of both human and canine visceral leishmaniasis
Source: PLoS Negl Trop Dis. 2020 Jul 27;14(7):e0008488. doi: 10.1371/journal.pntd.0008488 (PMC7410341; doi:10.1371/journal.pntd.0008488)

Supporting Figure S1. Schematic representation of the subcloning strategies used to generate the first set of chimeric proteins evaluated here (D1, D2 and D3). The figure illustrates the strategy used to generate D1, but identical strategies were used for the other two chimeric constructs. In all, the DNA fragments encoding the repeats were flanked by XhoI and SalI restriction sites and could be recovered by digestion with these enzymes, purified and ligated into the XhoI site of the recipient construct, with the correct orientation defined by restriction enzyme digestion/sequencing. Since SalI and XhoI have complementary overhang sequences, ligation could be achieved, but both sites were eliminated when ligated. A single 5' XhoI site remained then after the subcloning, at the fragment's 5' end, when in the correct orientation. This site could then be used in a subsequent subcloning step.

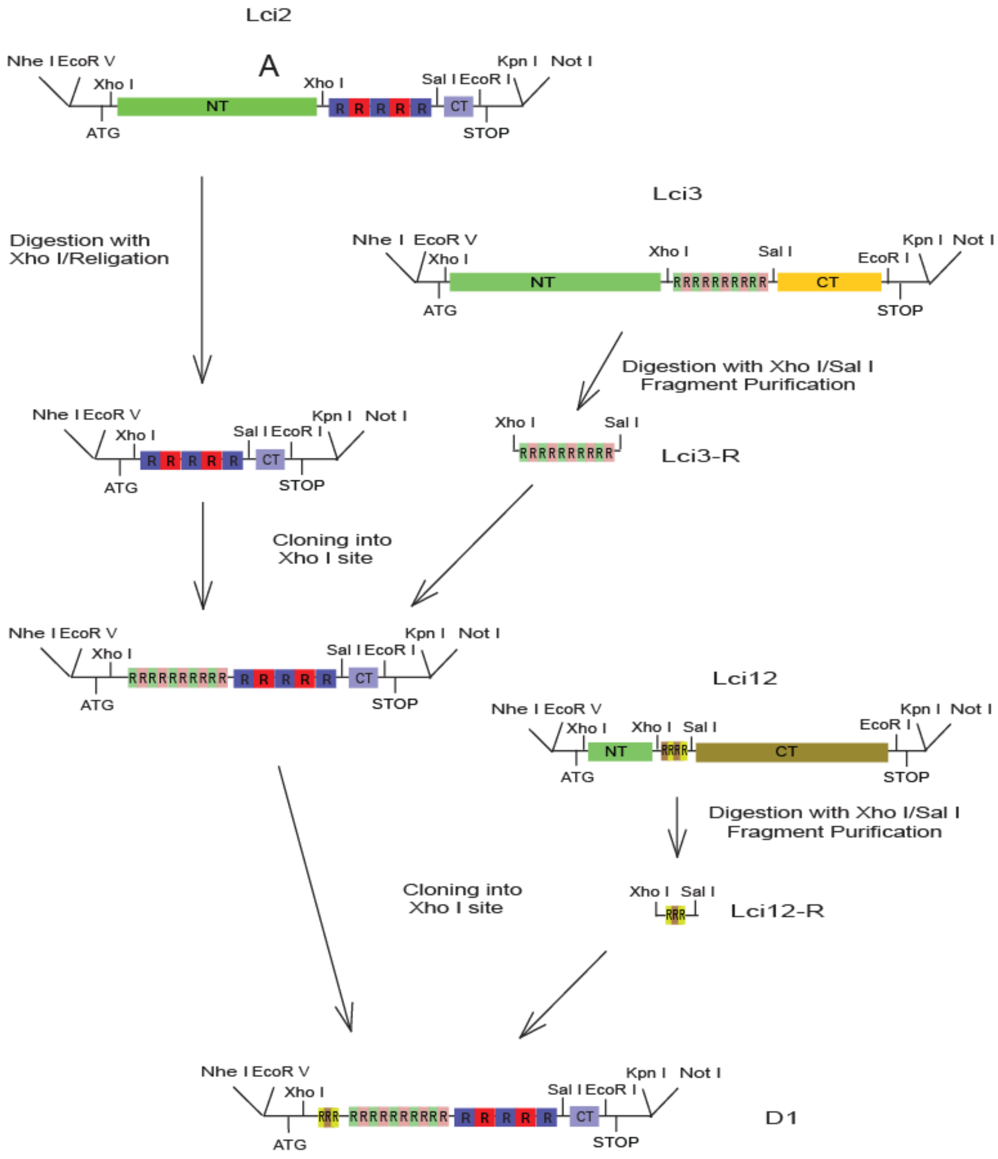

Supplement: S1 Fig — The figure illustrates the strategy used to generate D1, but identical strategies were used for the other two chimeric constructs. In all, the DNA fragments encoding the repeats were flanked by XhoI and SalI restriction sites and could be recovered by digestion with these enzymes, purified and ligated into the XhoI site of the recipient construct, with the correct orientation defined by restriction enzyme digestion/sequencing. Since SalI and XhoI have complementary overhang sequences, ligation could be achieved, but both sites were eliminated when ligated. A single 5’ XhoI site remained then after the subcloning, at the fragment’s 5’ end, when in the correct orientation. This site could then be used in a subsequent subcloning step. (PDF) [file pntd.0008488.s002.pdf]
