# Supplementary material for: Gene design, optimization of protein expression and preliminary evaluation of a new chimeric protein for the serological diagnosis of both human and canine visceral leishmaniasis
Source: PLoS Negl Trop Dis. 2020 Jul 27;14(7):e0008488. doi: 10.1371/journal.pntd.0008488 (PMC7410341; doi:10.1371/journal.pntd.0008488)

Supporting Figure S2. Schematic representation of the strategies used to modify the second set of chimeric proteins (Q1, Q2, Q3 and Q4). The figure illustrates the strategy used to generate Q1SX and Q1NN constructs from Q1, but identical strategies were used for the other constructs. In all, removal of selected DNA fragments was carried out by restriction enzyme digestions (SalI and XhoI for Q1SX or NcoI for Q1NN), followed by purification of the larger, plasmid plus Q1, fragment and religation. The full set of restriction enzymes used are listed in the Supporting Table S1.

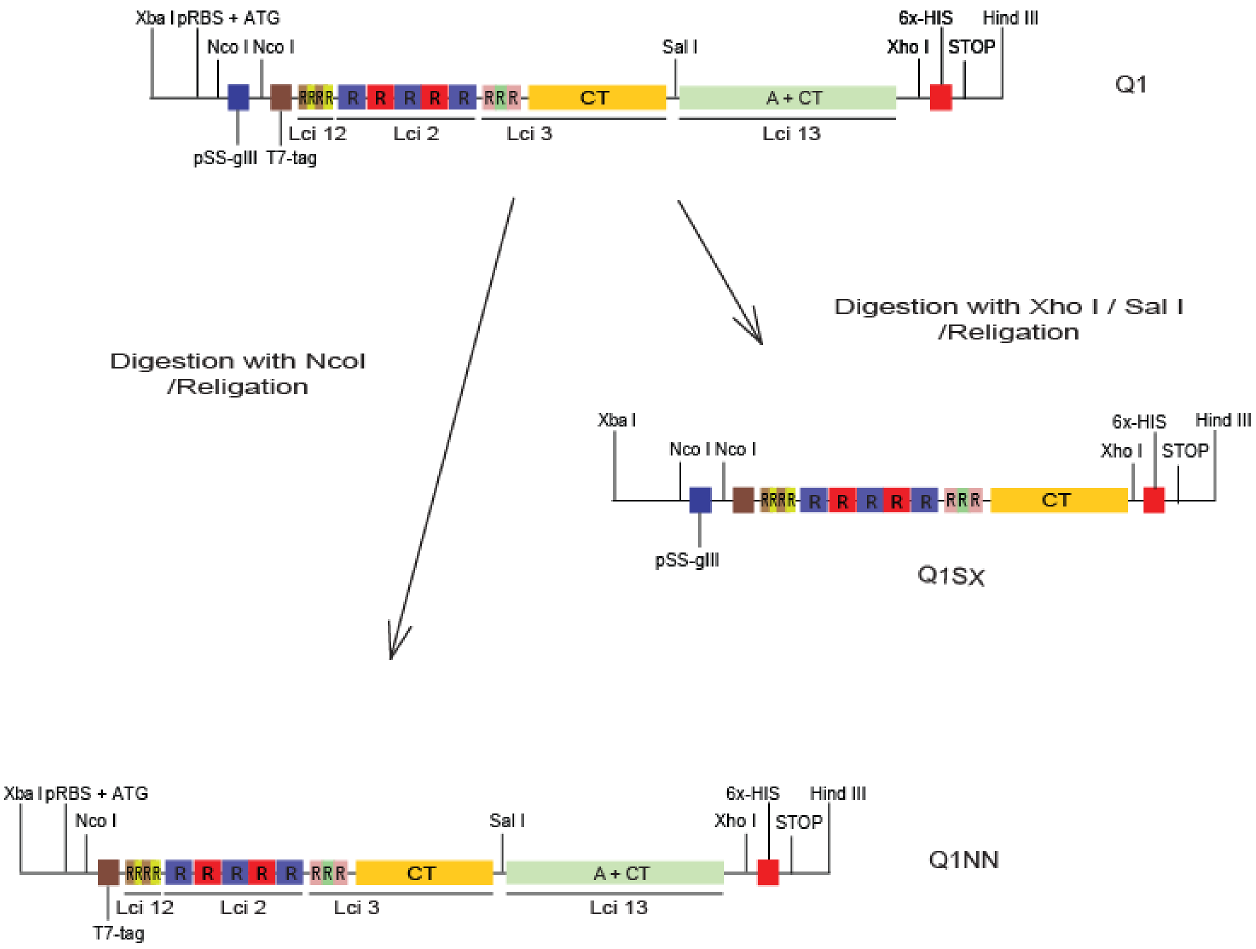

Supplement: S2 Fig — The figure illustrates the strategy used to generate Q1SX and Q1NN constructs from Q1, but identical strategies were used for the other constructs. In all, removal of selected DNA fragments was carried out by restriction enzyme digestions (SalI and XhoI for Q1SX or NcoI for Q1NN), followed by purification of the larger, plasmid plus Q1, fragment and religation. The full set of restriction enzymes used are listed in S1 Table. (PDF) [file pntd.0008488.s003.pdf]
