# Supplementary material for: Gene design, optimization of protein expression and preliminary evaluation of a new chimeric protein for the serological diagnosis of both human and canine visceral leishmaniasis
Source: PLoS Negl Trop Dis. 2020 Jul 27;14(7):e0008488. doi: 10.1371/journal.pntd.0008488 (PMC7410341; doi:10.1371/journal.pntd.0008488)
Supplement: S3 Fig — The sequence also shows the segment encoding the N-terminal His-Tag from the vector (in red). The Nhe I, Sal I, EcoR I and Not I restriction sites are underlined, while the two Xho I sites are underlined and in italic. The TGA stop codon is in pink. (PDF) [file pntd.0008488.s004.pdf]

Supporting Figure S3. Full length nucleotide sequence of the synthetic Lci2 gene after cloning within the pRSET vector. The sequence also shows the segment encoding the N-terminal His-Tag from the vector (in red). The Nhe I, Sal I, EcoR I and Not I restriction sites are underlined, while the two Xho I sites are underlined and in italic. The TGA stop codon is in pink.

**ATGCGGGGTTCTCATCATCATCATCATCATGGTATGGCTAGCGATATCACCATGGAA*CTCGAG*AT
GCACCCTAGCACCGTGCGGAGGGAAGCTGAAAGAGTCAAGGTTAGCGTGCGAGTGAGGCCCCTCA
ACGAACGAGAAAACAACGCCCCAGAGGGGACTAAGGTCACCGTGGCTGCCAAGCAAGCAGCTGCC
GTGGTCACGGTGAAAGTGTTGGGCGGCAGTAACAATAGCGGCGCAGCCGAAAGCATGGGACCGC
ACGGAGGGTTGCTCAGGATTTCCAGTTTGACCACGTGTTCTGGAGCGTCGAGACTCCCGACGCCT
GCGGCGCTACGCCAGCTACTCAGGCTGACGTCTTTAGGACCATCGGCTATCCCCTGGTCCAACAC
GCCTTTGACGGCTTCAATAGTTGTCTTTTCGCCTATGGGCAGACAGGATCAGGCAAAACCTACAC
TATGATGGGAGCTGACGTTAGTGCCCTGAGCGGCGAGGGGAACGGCGTCACTCCCAGAATCTGCC
TCGAAATCTTCGCCAGGAAGGCCAGCGTGGAGGCTCAAGGGCACAGTCGCTGGATCGTTGAGCTT
GGCTACGTTGAAGTGTACAACGAGAGAGTCAGCGACCTGTTGGGAAAGAGGAAGAAAGGAGTCAA
GGGGGGGGCGAGGAGGTGTACGTGGACGTGAGGGAACACCCTAGCAGAGGCGTCTTTCTGGAGG
GGCAAAGACTCGTGGAGGTGGGAAGCCTGGACGACGTTGTCAGGTTGATTGAGATCGGGAACGGC
GTCAGGCACACAGCCTCTACAAAGATGAATGACCGAAGCTCCCGTAGCCACGCTATCATCATGTT
GCTGCTCAGGGAGGAGCGGACAATGACTACTAAGTCAGGAGAAACAATCAGGACCGCAGGGAAGT
CCAGCAGGATGAATCTGGTGGACCTCGCCGGCAGCGAAAGGGTGGCTCAGTCCCAGGTGGAGGGG
CAACAGTTCAAGGAGGCCACACATATCAACTTGAGCCTGACAACCCTGGGCAGAGTGATCGACGT
GTTGGCTGATATGGCCACGAAGGGAGCCAAGGCTCAGTATTCAGTTGCTCCCTTTCGGGATAGTA
AACTTACGTTCATTCTGAAAGATTCACTTGGAGGGAATAGCAAGACCTTCATGATTGCCACCGTT
AGCCCTTCAGCCCTGAACTACGAGGAGACCTTGAGCACACTGAGATACGCCAGCCGTGCACGTGA
CATCGTGAACGTTGCTCAGGTCAACGAAGACCCACGAGCTAGGAGGATTCGTGAACTCGAAGAGC
AGATGGAAGATATGAGGCAGGCCATGGCTGGCGGAGACCCTGCCTACGTGAGCGAACTGAAGAAG
AAACTGGCACTGCTGGAGTCAGAGGCACAAAAGAGAGCAGCAGACCTCCAGGCCTTGGAACGGGA
GAGGGAGCACAATCAGGTCCAGGAAAGGCTGTTGAGAGCAACAGAAGCCGAGAAATCTGAGCTTG
AGTCCAGGGCAGCCGCCCTGCAGGAGGAGATGACCGCCACCCGAAGGCAGGCCGATAAGATGCAA
GCACTGAACCTCAGGCTGAAGGAGGAGCAGGCACGGAAAGAGCGTGAACTGTTGAAAGAGATGGC
CAAGAAGGACGCTGCCCTGAGCAAGGTGAGGCGCAGAAAGGACGCTGAGATCGCAAGCGAGAGGG
AGAAGCTGGAGTCCACCGTGGCACAGCTCGAACGCGAGCAGAGAGAGAGGGAGGTGGCACTTGAC
GCCTTGCAGACCCACCAACGCAAGTTGCAAGAAGCCTTGGAAAGTAGCGAGCGGACTGCAGCCGA
AAGGGATCAGCTGTTGCAGCAGCTCACTGAACTCCAGAGCGAGCGGACCCAACTGAGCCAGGTGG
TTACTGATAGGGAGCGACTGACCAGGGACCTGCAACGGATTCAGTACGAGTACGGAGAGACAGAG
CTGGCAAGGGACGTGGCCCTGTGTGCCGCCCAGGAGATGGAAGCTCGCTATCACGCAGCAGTGTT
TCACCTGCAAACACTGCTGGAGCTGGCCACTGAGTGGGAAGATGCCCTGAGGGAGAGAGCACTGG
CCGAGCGTGACGAAGCAGCTGCTGCCGAACTGGATGCTGCTGCCAGCACGTCCCAGAACGCTCGG
GAGTCAGCCTGTGAGAGACTGACAAGT*CTCGAG*CTTGAAGAGAAACTGAGGGGAACTGAAGCCAG
AGCTGCCGAACTCGCCGCCAGGCTGAAGGCCATTGCTGCCATGAAAGCAAGCATGGTGCAGGAAA
GGGAGTCCGCACGCGACGCACTGGAAGAAAAGCTGAGGGGCAGCGAGGTGAGGGCCGCAGAGCTC
GCAGCCAGACTCAAAGCCGCAGTGGCAGCCAAAAGCAGCGCAGAACAGGATAGAGAAAACACGAG
AGCCACCCTGGAACAGAGACTGAGGGAGAGTGAGGAAAGGGCCGCAGAGCTGGCCAGTCAGCTGG
AAGCAGCCGCAGCCGCAAAGAGCAGCGCAGAGCAGGACAGGGAAAACACACGAGCAGCCCTGGAG
GAAAAGCTGAGGGGATCAGAGGAGAGGGCTGCAGAGCTGGGCACCCGAGTCAAGGCCAGCAGCGC
CGCAAAGGCCCTTGCCGAGCAGGAACGCGATAGGATTAGGGCTGCTTTGGAAGAGAAACTGAGGG
ATAGCGAGGCCAGAGCTGCCGAACTGACCACCAAGCTGGAGGCCACTGTGGCCGCCAAATCAAGT
GCCGAGCAAGAGAGAGAGAACATCAAAGTGGCAGTCGACCTGGAGGAGGAGCTGGTGGACGCACG
AGCCAAACTGGCTGGCATGGAAGCCAGCCTGAAAGAAAGCAAGCTGGAGTTTGAAGGGAGGGTGG
GCGAACTGGAGGGAGAGTGCGAGAAACTCAGGAACGACAAAGTGCGATACGCCAAGAAGGTCCAG
TCTCTGGAGTATCAGATGCGTATTGACGAGGCCAGGCTCAAAGCAAGGAGGGATGCTGTGCACAG
GAAAGAAGAATTCTGATAAGGTACCGCGGCCGC**
