# Supplementary material for: Gene design, optimization of protein expression and preliminary evaluation of a new chimeric protein for the serological diagnosis of both human and canine visceral leishmaniasis
Source: PLoS Negl Trop Dis. 2020 Jul 27;14(7):e0008488. doi: 10.1371/journal.pntd.0008488 (PMC7410341; doi:10.1371/journal.pntd.0008488)
Supplement: S4 Fig — The sequence also shows the N-terminal His-Tag encoded by the vector (in red) and elements introduced during the synthesis and cloning procedures in purple. The N-terminus, the region encoding the repeats and the C-terminal segments are in green, orange and blue, respectively. (PDF) [file pntd.0008488.s005.pdf]

Supporting Figure S4. Full length amino acid sequence of the synthetic Lci2 recombinant protein after cloning within the pRSET vector. The sequence also shows the N-terminal His-Tag encoded by the vector (in red) and elements introduced during the synthesis and cloning procedures in purple. The N-terminus, the region encoding the repeats and the C-terminal segments are in green, orange and blue, respectively.

MRGSHHHHHHGMASDITMELEMHPSTVRREAERVKVSVRVRPLNERENNAPEGTKVTVAAKQAA
AVVTVKVLGGSNNSGAAESMGTARRVAQDFQFDHVFWSVETPDACGATPATQADVFRTIGYPLV
QHAFDGFNSCLFAYGQTGSGKTYTMMGADVSALSGEGNGVTPRICLEIFARKASVEAQGHSRWI
VELGYVEVYNERVSDLLGKRKKGVKGGGEEVYVDVREHPSRGVFLEGQRLVEVGSLDDVVRLIE
IGNGVRHTASTKMNDRSSRSHAIIMLLLREERTMTTKSGETIRTAGKSSRMNLVDLAGSERVAQ
SQVEGQQFKEATHINLSLTTLGRVIDVLADMATKGAKAQYSVAPFRDSKLTFILKDSLGGNSKT
FMIATVSPSALNYEETLSTLRYASRARDIVNVAQVNEDPRARRIRELEEQMEDMRQAMAGGDPA
YVSELKKKLALLESEAQKRAADLQALEREREHNQVQERLLRATEAEKSELESRAAALQEEMTAT
RRQADKMQALNLRLKEEQARKERELLKEMAKKDAALSKVRRRKDAEIASEREKLESTVAQLERE
QREREVALDALQTHQRKLQEALESSERTAAERDQLLQQLTELQSERTQLSQVVTDRERLTRDLQ
RIQYEYGETELARDVALCAAQEMEARYHAAVFHLQTLLELATEWEDALRERALAERDEAAAAEL
DAAASTSQNARESACERLTSLELEEKLRGTEARAAELAARLKAIAAMKASMVQERESARDALEE
KLRGSEVRAAELAARLKAAVAAKSSAEQDRENTRATLEQRLRESEERAAELASQLEAAAAAKSS
AEQDRENTRAALEEKLRGSEERAAELGTRVKASSAAKALAEQERDRIRAALEEKLRDSEARAAE
LTTKLEATVAAKSSAEQERENIKVAVDLEEELVDARAKLAGMEASLKESKLEFEGRVGELEGEC
EKLRNDKVRYAKKVQSLEYQMRIDEARLKARRDAVHRKEEF
