# Supplementary material for: Gene design, optimization of protein expression and preliminary evaluation of a new chimeric protein for the serological diagnosis of both human and canine visceral leishmaniasis
Source: PLoS Negl Trop Dis. 2020 Jul 27;14(7):e0008488. doi: 10.1371/journal.pntd.0008488 (PMC7410341; doi:10.1371/journal.pntd.0008488)
Supplement: S5 Fig — The sequence also shows the segment encoding the N-terminal His-Tag from the vector (in red). The Nhe I, Sal I, EcoR I and Not I restriction sites are underlined, while the two Xho I sites are underlined and in italic. The TGA stop codon is in pink. (PDF) [file pntd.0008488.s006.pdf]

Supporting Figure S5. Full length nucleotide sequence of the synthetic Lci3 gene after cloning within the pRSET vector. The sequence also shows the segment encoding the N-terminal His-Tag from the vector (in red). The Nhe I, Sal I, EcoR I and Not I restriction sites are underlined, while the two Xho I sites are underlined and in italic. The TGA stop codon is in pink.

ATGCGGGGTTCTCATCATCATCATCATCATGGTATGGCTAGCGATATCACCATGGAG*CTCGAG*A
TGTCCTTCCTGTCTAACATCAATCCTGCCTTTCCTCAACTTCAAGCTGGCCAGGTGGTGGCCTA
CGACTACCTCCACGCCGCTAAGACTTGGCAGTGGACACTGGGACCGTGCGAGAGATCAAGGAT
TACACAGCAGTGGTGCAGCAGTGGGACTGCACACGGGAGACATCGACACACTGCGGAGTATCC
TGCTCAAGGAGGTGGACACTGAGAACGGGAGGATGAAGAACTATCACGATATGCTTGCCATCGC
AAGGGAGAAGCTGGCCAGCATTAGGCGTAGCAATGAAGACAGGGTGAGCCACGTGCGTGGCCAC
TTCGACAAAGCAAGGGAGAAAGTGGAACTGATTGACGAAGTGGACCTGAGGAAAGTCACTGCTC
AAGCCGCTCCAAGCCCTGTGGCTGTGGCCGTCCTGAAAGCCGTGTGGGCTGTGGCCAAGTGCGA
CCCCACCGCCGTCGAGTTCTACGAGTGGGCTGACGTCCAGCTGGAGTATCGGAAGCCCGCCGCC
CTGGACGAGATCGCTAAGACCGACGTGCTGGCAAAGCTCTATCCCAGCGCTGAGAGCCTGCAGC
AGTCCCTGGAGCAGGACCCTAAGCTCAACTACAAAGCCGCTGCCAGGGATAGTCCCGTCGTTGC
CTCCCTGCACGCTTGGGTGATTACAGCCCTGGCCTATCAGCAGGCTTACAACCTGCTGGCTCAC
GACAAAAGAATCCAGGAACAGAATGACGCCATTGCAGCAGCAATTGCCGGAATGAAAGCCTGTA
GGGCAAAGATCGCCAAGCTGAAGGACGAGCTGTCTAGCAAAGACACAGCAGCCCTCCCAGGGCA
GGTGACTAGCTTCACTAGGACAAGCGTGCTGGTGACTATCCCTCTGAGCGCCGTGATTAGTCCT
GTCAACGTTGACACAGGCGTGAAAGGCTGCGTCCTGACAAAGGACGAGGTGGAGCAGATCCTGT
TTGAAGCCAAGGCCACCAGGTTCCAACTCAAGTCTAGGATGAATAGCATTGCCTGCCGTTACGT
GGAAGCAGCCGCTGAGCTGCACACCCTGAGCCTCTACACCGCTGAATTGGAAAAGAAGAGGCTG
TACCTGCAGGAACACTACTTCTCTTCTATCATCCGGTCCGGCGAAGCTGAGGCCAAGTGGGCAC
GGACCGAAGATACCCAAAAGGAGATCGATAGGCTCAACGCACTGGTGGCTGAGCTCCAAAAGCA
CGACGAGAGGTGGGAACCCGATTACGAGGCAGTCAGCGTTGCCACCAGCCACGTCAAGAAGTAT
CCCGGAGCCGAGTGGGCCTATCTGATCGCTGAGAGGTTCGAGGAGGTGAGAGCTGCCTTCGCCT
CTGACACTGCTCTTGCCGTGCACGTGGACCCCAACTTCGTGCAGCACATCAAGTTTACCCCAGA
GTGCGACCAGCTGTGCGTGCGCTGCGAAATCACACACCCTGCCAAGATGACCGGAACCGAGGTG
GACGAGAGGATTAGTCAGATGCCTACACGACTGATGAATTACATCTACAAGAATAGAGACGCTC
CAAAGACCGGACTGGACCGAGCAGTTGCCGACATCTGCAATGCACTGGGGATCGACGACCACAA
ATTCGCTGGCTTGGGCTTTGACGAGTTCGTGACCCAGCTGGCTGGCTTCGACTACCTGGGCGAC
AAAGATGCCTACGAAAGCGAGATCGGAGATCTTCTGATGCTGCTGGACAAGATCAACAACGAGA
ATCGCAGCCTCCAGTACACGCTGGAGAAGAGTGCTGAAAGGTTCAAGAAACAGGCCGCTGTTCT
GCAGCGAGATCAAGACGCTCTTACGCTGAGAAACGCTGACCTGGCTGACGAGATCGATAGGCTG
CAAAACCTGGTGGAGAAGCTGAAAGACCTGGCAGACACTCAAGGAGCCCAGCTGGAACACTACC
ACATGCAGCACCAGCAAGCTCAGCAGCTGCGTGCTCACAGAAACCTGAGTCCCATCCCCCCAAC
CGCAGAAGAGCCACTGTACGCTGTTACCATCGACGAGCTCAACGCTCAAAAGGCCCTGTGTGAC
AAAGAAAAGCAGAGGGCCGACGCCCTGCAGAAACAACTCGACGACAAAGAGCTGGCTCTGAATC
AGCTGCAAAGCCAGCTGAGGGAAGTTAGGTGCAGGAACAACGACTTGGATCAGCAGCTGCAGCT
GAGCGCTGAACTGAGCGAGAAGCAGAAGCAGATCCTCACTGCCTTCCACCAGAAGAGAAGAAGC
GCCCACGACGCAAGGGCCGACGAACCCGAACTGGCAGCAGCCGACGGCGTGTCAACCCGAAACG
CCAGTGCCAGAAGTAGGGGCAGACCTCCCGCCCACATTACAACTGCTGCCGAGCCTTTCGACCC
CGTGACTATCGCCGCAGACCCTCTTTACGCCGTTACACTGGACGAGTACAAAGCAAAACAGACG
GCCCTGAATAGTGCCAAGGAGGAAGTGCAAAGGCTGGTGGGC*CTCGAG*GAGCTGCAGAAAGCCC
AGGAGGACGGCGAACGTCAAAAGGCAGACAATAGACAGCTGGCCTCAGACAACGAGAGACTGGC
CACCGAGCTGGAAAGAGCTCAGGAGGAAGCAGAGCGCCTGGCCGGAGACCTGGAGAAAGCAGAA
GAAGAGGCTGAGCGACTTGCAGGCGACCTGGAGAAGGCCCAGGAAGAGGCAGAAACACTGGCTG
GGGAGCTCCAAAAGGCCCAGGAGGACGGGGAACGTCAAAAGGCAGACAATCGGCAGCTGGCCTC
AGACAACGAAAGGCTGGCCACTGAGCTGGAGAGAGCCCAGGAAGAGGCTGAAAGGCTGGCAGGC
GACCTGGAGAAAGCTGAGGAGGAGGCAGAAAGACTGGCAGGCGACCTGGAAAAAGCCCAAGAGG
AAGCTGAGACGCTGGCTGGCGTCGACGAGCTGGCTGACAAGGACCCAGAATTGGCCGCCTTTAG

GGAAAAGCGCAGGGCCGCTCACGGAGCCAGAGCAGACGAACCCGAGCTGGCTGCTGCCGACGGG
ATTAGCACACGCAATGCCAGGGCCGGAAGCCGTGGACGTCCAGCCGCACAGATCAATCCCGCTG
CTGAAGCCGTGGATCCCGTGACTATCGCAGCTGAGCCACTGTACGCCGTGACCCTCGACGAATA
CAAGGCCAAACAGACCGCACTGGAAAACGCAGTTGAAGTGGCCTGCGCAGCCGAAGAGACTGTG
AAAGAGAAACTGAGGGAGAACAGCGACCTGATGGTGGAGCTGGAAAAGGTGCGTGACCAGGCTT
ACGAGATGGATAGGAGGAGGCAAGAAGACGGAGCCGCCATGGAAGGGGAGCTGCTGGTTGTGCT
GATGGAGCTCAAGAAACTCAAGGGAATCAACGACGCCCTGCTGGCTGTGCTTAGGGACAAAGAG
TGTGAGGTGAAAGAGCTTCGATACCACAACGAGTTGTGGGTTGACCCAACGGGAGACAAGAAGC
AGGTGGTGACGAGGCACACTAAGATCTTTGACGGCAATTGGGAGAGGATTGTGCGAGAACGACC
CGAAGGGCTGTTCGCAGCCTTTGTGATCGATAGCAGTAACGCCTGCCACGTCCCTGGGGACAAC
ATCAAACAGGTGTCTTTTGACCACGACGAATTCTGATAAGGTACCGCGGCCGC
