# Supplementary material for: Gene design, optimization of protein expression and preliminary evaluation of a new chimeric protein for the serological diagnosis of both human and canine visceral leishmaniasis
Source: PLoS Negl Trop Dis. 2020 Jul 27;14(7):e0008488. doi: 10.1371/journal.pntd.0008488 (PMC7410341; doi:10.1371/journal.pntd.0008488)
Supplement: S7 Fig — The sequence also shows the segment encoding the N-terminal His-Tag from the vector (in red). The Nhe I, Sal I, EcoR I and Not I restriction sites are underlined, while the two Xho I sites are underlined and in italic. The TGA stop codon is in pink. (PDF) [file pntd.0008488.s008.pdf]

Supporting Figure S7. Full length nucleotide sequence of the synthetic Lci12 gene after cloning within the pRSET vector. The sequence also shows the segment encoding the N-terminal His-Tag from the vector (in red). The Nhe I, Sal I, EcoR I and Not I restriction sites are underlined, while the two Xho I sites are underlined and in italic. The TGA stop codon is in pink.

**ATGCGGGGTTCTCATCATCATCATCATCATGGTATGGCTAGCGATATCACCATGGAA*CTCGAG*A
TGAGCACCGACAACGACATTGAGAGGCAGATCATGATGGAGATGGAGGCAGAGATCAGCAGGTC
ACAGGGAAACAGGCGTGACCCCTACACCAATCCCCCCCCCTTCGAGCTGAGCTTCATCGAGGAT
GACCCTATGGAGGCAGCCAGGAAGGCAGAAGTGGACAGGATCCAGAGAGAAATCGAGGAGAGGC
TGAGGAGGAAACAACAGCAGAAGCAGCGAGACAGCCTGGAGCTGAGTCCTAACTGCCCCGCTAA
CGAGGGAGAGATGAGTGCCGTCTACGACAGCCTGCAACAGCCCCAAAACGCCAGCTGGCCAGTG
GGAGCTCACGATAGGCACGTGGTGAGGGCAAGCCTGAGCTCCGAGAGCGAGAATAGAGAGAAAG
CTGAAGAGGCCAGCCGACTGAGGGACGACGAGGCTGCCATGACACGGAGGGAAACTGACGAAGA
GGCCAGGATCGATATGGAAGTCCAGGTGAGGAGAAGTGCCGAGATTCAGGCACTGCGTGAG*CTC
GAG*GCCGAGGAACAGGCCAGGAGGGAGGCTGAAGAGCAGGCCAGACGCGTCGCCGAGGAACAGG
CCAGGAGGGAGGCAGAGGAGCAAGCCAGGAGAGAGGTCGACCAGGCCGGAGGCCTGGAGGAGGT
GTTGCGAGAGGCCAGGGCTGTCGTGATGGGCGAGTTCTCCGAGAAGCTGAAGCAACACAGTGCT
GTGGCTGCCTTCGTGAGTGACACAGACGAGAAACCCGTGAATAGCGCCTCAGCAACTCACAGAG
GGGACGCCAGGTGGCAGAACGAGTACACTGAACAAGGGGGAACTGGAGCAGACGCCGAGGAAGA
GCATGGCGTGCAACAGCACGATAGCAGGTGCTGCCGCTACCGAACGAGCAGTCCAAGCGTGAGT
GACAGAGATATGAGATCAGAACGTTCTACTAACAGCAAAGACTCAGCCAGCGAAACAGTGAGCA
GGTATAGCCTGAGCACCCTTGAGGCCATTAGGAACGACAACGGGATCCTGTCCCGTACCGAGGA
GGAGGTGTACTACGTCCCCAGGGTGACCAAGGAAGCCCCATTCGAGAGCTTTGAAGAGGCCTTG
AGTGCCGAACTGAAAGCACACGGGCTGACTGAGGACGCCATCCGGAGGAGCTGCATTGAGGTTC
ACAGGTACGGAACCATCAGGGAGAGCGGCAAGTGCCTGTTCCCACCCCGGGAGGTCACGGGCGA
GGTCCCCGCACACGGAAAGGTGCAGCTGGGCTTCTTCAGCGCTAAGCAAACAATCATTGCCCTG
CAGAGGCCCCTTAGGAAGCCAAACGCCGACCGAGAACGACCCTGCGAGCCAGGCGAACGAAGCC
TGTCAACCCTCAAGTGTTACTTTGAGAGCGAAGTGCTGAGCGACCATACGCTGACCGTGGACGA
CGAAGACTATCCCCATAACGCCCCTACCGAACGACTCCTGACCAAGGCCCAGCTGATGAGAGGG
GACAACGCCATGGTGAGGAAAGCTGTTAGCCAGATCAGCTATGGGGACCCAATTCAGGTGTGGG
AAAGAGCCCAGCAGAATAGCACCGCTGCCGACGAGGCTGCCACCGAGAGCACTGCCGCCAATCT
GTGGGTGAGCGACATTGACACCCGGAAGCCTGTGCCTGCCATGAGGACGTTTACCGGCGGATTC
GTCTACTGTATCAAGGCAACAAAGATCAGCAATAGGGTGATCGAACTGCACGGAGCCAGCACAG
ACCCTTTGGTGATCGCCGCAGCCCTTTACTCATGGACGGAAAGACAGAAAGTGAAAGTGAGCGA
GACCTTCTACTTCGATAGCGAATTGGACATCTTCTACCCTCAGAAGGAGAGGAGTGAGCTGGCT
AAGAAGAATCAGGTCGTGGCATTCGTGCCAAATGAGTTTAAAGGCACACTGCATCTCGTCATGA
GGGTGTACCGTCCCTGTTGCGAGGAGTATGATACTTACGTGGACCTTTACTCTAGGGCAGATAG
GTACAAACAGATTCACGTGGCCCCTATGAAGCAGGAGACCCTGCTGTTGACTCAGGTGAGCGAC
GTGCTGGAGGAACTGGGCTGGAATAGCGTTCCCCTGCAAGACGAGGCAAACCACCTGCTGCCCA
GGGTGGCCGTGGACAGACTGTATAGGAAAGCCTTCTCAAATGAGGACGTGTTCAAAGTGATGAA
AGATGAGAGGTGGAGAGGCGCACAGAAAGCCCTGCCTGTGGATATGGTGTTCTCAATCAGCGAC
CTCAGCAGGCATGAGGTCGCCTTCCCTTCTGATCATCCCGAAACTCCCCCTGAGGAAAACGAAA
GCAAGGTTAGCCTGCTGGATCCCGACCTGCCTGGCGCTAGGCCTGTGATCTACAGGTACTCACC
CTGCTGCATCCCTATCCTGAATAGCGGCTATTTCACGACCTACAACAACGTGTACTACTTTTCT
GTGAGCAGGCTGAAAGTGATGTACGCCGGCTTCGTGAGGAGTATCCCAGCAAGCCACCATACCT
ACGTGTTCCAACTGTGTGTGAAAGACAAGGATGACGGACTTAGCGAGGAAGGGGCAATCAGATG
CATCTACGGGAGAGGCCTGTCAAACCTGAGCATGGAGACCACCGCCTGGAGCTCTTCTGTGCAC
AATAGCAATGACATGGTGCTGTCTGATGAGTTCAAGCTGCAAGAATTCTGATAAGGTACCGCGG
CCGC**
