# Supplementary material for: Gene design, optimization of protein expression and preliminary evaluation of a new chimeric protein for the serological diagnosis of both human and canine visceral leishmaniasis
Source: PLoS Negl Trop Dis. 2020 Jul 27;14(7):e0008488. doi: 10.1371/journal.pntd.0008488 (PMC7410341; doi:10.1371/journal.pntd.0008488)
Supplement: S9 Fig — The sequence also shows (in black) the segments introduced by the vector, with the region encoding the his-tag in red and elements introduced by the cloning procedures in purple and the stop codon in pink. The Xho I and Eco RI flanking sites are underlined. Fragments encoding the repeats from Lci12, Lci2 and Lci3 are in green, orange and blue, respectively. The Lci3 fragment lacking repeats is in brown. (PDF) [file pntd.0008488.s010.pdf]

Supporting Figure S9. Full length nucleotide sequence of the recombinant D2 gene within the pRSET vector. The sequence also shows (in black) the segments introduced by the vector, with the region encoding the his-tag in red and elements introduced during the synthesis and cloning procedures in purple and the stop codon in pink. The Xho I and Eco RI flanking sites are underlined. Fragments encoding the repeats from Lci12, Lci2 and Lci3 are in green, orange and blue, respectively. The Lci3 fragment lacking repeats is in brown.

ATGCGGGGTTCTCATCATCATCATCATCATGGTATGGCTAGCATGACTGGTGGACAGCAAATGG
GTCGGGATCTGTACGACGATGACGATAAGGATCGATGGGGATCCGAGCTCGAGGCCGAGGAACA
GGCCAGGAGGGAGGCTGAAGAGCAGGCCAGACGCGTCGCCGAGGAACAGGCCAGGAGGGAGGCA
GAGGAGCAAGCCAGGAGAGAGGTCGAGCTTGAAGAGAAACTGAGGGGAACTGAAGCCAGAGCTG
CCGAACTCGCCGCCAGGCTGAAGGCCATTGCTGCCATGAAAGCAAGCATGGTGCAGGAAAGGGA
GTCCGCACGCGACGCACTGGAAGAAAAGCTGAGGGGCAGCGAGGTGAGGGCCGCAGAGCTCGCA
GCCAGACTCAAAGCCGCAGTGGCAGCCAAAAGCAGCGCAGAACAGGATAGAGAAAACACGAGAG
CCACCCTGGAACAGAGACTGAGGGAGAGTGAGGAAAGGGCCGCAGAGCTGGCCAGTCAGCTGGA
AGCAGCCGCAGCCGCAAAGAGCAGCGCAGAGCAGGACAGGGAAAACACACGAGCAGCCCTGGAG
GAAAAGCTGAGGGGATCAGAGGAGAGGGCTGCAGAGCTGGGCACCCGAGTCAAGGCCAGCAGCG
CCGCAAAGGCCCTTGCCGAGCAGGAACGCGATAGGATTAGGGCTGCTTTGGAAGAGAAACTGAG
GGATAGCGAGGCCAGAGCTGCCGAACTGACCACCAAGCTGGAGGCCACTGTGGCCGCCAAATCA
AGTGCCGAGCAAGAGAGAGAGAACATCAAAGTGGCAGTCGAGGAGCTGCAGAAAGCCCAGGAGG
ACGGCGAACGTCAAAAGGCAGACAATAGACAGCTGGCCTCAGACAACGAGAGACTGGCCACCGA
GCTGGAAAGAGCTCAGGAGGAAGCAGAGCGCCTGGCCGGAGACCTGGAGAAAGCAGAAGAAGAG
GCTGAGCGACTTGCAGGCGACCTGGAGAAGGCCCAGGAAGAGGCAGAAACACTGGCTGGGGAGC
TCCAAAAGGCCCAGGAGGACGGGGAACGTCAAAAGGCAGACAATCGGCAGCTGGCCTCAGACAA
CGAAAGGCTGGCCACTGAGCTGGAGAGAGCCCAGGAAGAGGCTGAAAGGCTGGCAGGCGACCTG
GAGAAAGCTGAGGAGGAGGCAGAAAGACTGGCAGGCGACCTGGAAAAAGCCCAAGAGGAAGCTG
AGACGCTGGCTGGCGTCGACGAGCTGGCTGACAAGGACCCAGAATTGGCCGCCTTTAGGGAAAA
GCGCAGGGCCGCTCACGGAGCCAGAGCAGACGAACCCGAGCTGGCTGCTGCCGACGGGATTAGC
ACACGCAATGCCAGGGCCGGAAGCCGTGGACGTCCAGCCGCACAGATCAATCCCGCTGCTGAAG
CCGTGGATCCCGTGACTATCGCAGCTGAGCCACTGTACGCCGTGACCCTCGACGAATACAAGGC
CAAACAGACCGCACTGGAAAACGCAGTTGAAGTGGCCTGCGCAGCCGAAGAGACTGTGAAAGAG
AAACTGAGGGAGAACAGCGACCTGATGGTGGAGCTGGAAAAGGTGCGTGACCAGGCTTACGAGA
TGGATAGGAGGAGGCAAGAAGACGGAGCCGCCATGGAAGGGGAGCTGCTGGTTGTGCTGATGGA
GCTCAAGAAACTCAAGGGAATCAACGACGCCCTGCTGGCTGTGCTTAGGGACAAAGAGTGTGAG
GTGAAAGAGCTTCGATACCACAACGAGTTGTGGGTTGACCCAACGGGAGACAAGAAGCAGGTGG
TGACGAGGCACACTAAGATCTTTGACGGCAATTGGGAGAGGATTGTGCGAGAACGACCCGAAGG
GCTGTTCGCAGCCTTTGTGATCGATAGCAGTAACGCCTGCCACGTCCCTGGGGACAACATCAAA
CAGGTGTCTTTTGACCACGACGAATTCGAAGCTTGA
