# Supplementary material for: Gene design, optimization of protein expression and preliminary evaluation of a new chimeric protein for the serological diagnosis of both human and canine visceral leishmaniasis
Source: PLoS Negl Trop Dis. 2020 Jul 27;14(7):e0008488. doi: 10.1371/journal.pntd.0008488 (PMC7410341; doi:10.1371/journal.pntd.0008488)
Supplement: S10 Fig — The sequence also shows the N and C-terminal segments encoded by the vector (in black) with the histidine tag highlighted (in red) and elements introduced during the synthesis and cloning procedures in purple. The fragments encoding the repeats from Lci12, Lci2 and Lci3 are in green, orange and blue, respectively. The Lci3 fragment lacking repeats is in brown. (PDF) [file pntd.0008488.s011.pdf]

**Supporting Figure S10. Full length amino acid sequence of the recombinant D2 protein within the pRSET vector.** The sequence also shows the N and C-terminal segments encoded by the vector (in black) with the histidine tag highlighted (in red) and elements introduced during the synthesis and cloning procedures in purple. The fragments encoding the repeats from Lci12, Lci2 and Lci3 are in green, orange and blue, respectively. The Lci3 fragment lacking repeats is in brown.

MRGSHHHHHHGMASMTGGQQMGRDLYDDDDKDRWGSELEAEEQARREAEEQARRVAEEQARREA
EEQARREVELEEKLRGTEARAAELAARLKAIAAMKASMVQERESARDALEEKLRGSEVRAAELA
ARLKAAVAAKSSAEQDRENTRATLEQRLRESEERAAELASQLEAAAAAKSSAEQDRENTRAALE
EKLRGSEERAAELGTRVKASSAAKALAEQERDRIRAALEEKLRDSEARAAELTTKLEATVAAKS
SAEQERENIKVAVEELQKAQEDGERQKADNRQLASDNERLATELERAQEEAERLAGDLEKAEEE
AERLAGDLEKAQEEAETLAGELQKAQEDGERQKADNRQLASDNERLATELERAQEEAERLAGDL
EKAEEEAERLAGDLEKAQEEAETLAGVDELADKDPELAAFREKRRAAHGARADEPELAAADGIS
TRNARAGSRGRPAAQINPAAEAVDPVTIAAEPLYAVTLDEYKAKQTALENAVEVACAAEETVKE
KLRENSDLMVELEKVRDQAYEMDRRRQEDGAAMEGELLVVLMELKKLKGINDALLAVLRDKECE
VKELRYHNELWVDPTGDKKQVVTRHTKIFDGNWERIVRERPEGLFAAFVIDSSNACHVPGDNIK
QVSFDHDEFEA
