# Supplementary material for: Gene design, optimization of protein expression and preliminary evaluation of a new chimeric protein for the serological diagnosis of both human and canine visceral leishmaniasis
Source: PLoS Negl Trop Dis. 2020 Jul 27;14(7):e0008488. doi: 10.1371/journal.pntd.0008488 (PMC7410341; doi:10.1371/journal.pntd.0008488)
Supplement: S11 Fig — The sequence also shows the flanking XbaI and HindIII sites, underlined. The segment encoding the pSS-gIII peptide, flanked by the two NcoI sites, is in pink, while the segment encoding the T7 tag epitope is in light blue. Segments encoding the repeats from Lci12, Lci2 and Lci3 are in green, orange and dark blue, respectively, while the fragment encoding the His-Tag is in red. (PDF) [file pntd.0008488.s012.pdf]

Supporting Figure S11. Full length nucleotide sequence of the recombinant Q1SX gene. The sequence also shows the flanking XbaI and HindIII sites, underlined. The segment encoding the pSS-gIII peptide, flanked by the two NcoI sites, is in pink, while the segment encoding the T7 tag epitope is in light blue. Segments encoding the regions from Lci12, Lci2 and Lci3 are in green, orange and dark blue, respectively, while the fragment encoding the His-Tag is in red.

TCTAGAAGGAGGTAAACCATGGCAAAAAAACTGCTGTTCGCGATTCCGCTGGTGGTGCCGTTCTATAG
CCATACCATGGCTAGCATGACTGGTGGACAGCAAATGGGTCGGATGATCGAGGCCGAGGAACAGGCCA
GGAGGGAGGCTGAAGAGCAGGCCAGACGCGTCGCCGAGGAACAGGCCAGGAGGGAGGCAGAGGAGCAA
GCCAGGAGAGAGGTCGAGCTTGAAGAGAAACTGAGGGGAACTGAAGCCAGAGCTGCCGAACTCGCCGC
CAGGCTGAAGGCCATTGCTGCCATGAAAGCAAGCATGGTGCAGGAAAGGGAGTCCGCACGCGACGCAC
TGGAAGAAAAGCTGAGGGGCAGCGAGGTGAGGGCCGCAGAGCTCGCAGCCAGACTCAAAGCCGCAGTG
GCAGCCAAAAGCAGCGCAGAACAGGATAGAGAAAACACGAGAGCCACCCTGGAACAGAGACTGAGGGA
GAGTGAGGAAAGGGCCGCAGAGCTGGCCAGTCAGCTGGAAGCAGCCGCAGCCGCAAAGAGCAGCGCAG
AGCAGGACAGGGAAAACACACGAGCAGCCCTGGAGGAAAAGCTGAGGGGATCAGAGGAGAGGGCTGCA
GAGCTGGGCACCCGAGTCAAGGCCAGCAGCGCCGCAAAGGCCCTTGCCGAGCAGGAACGCGATAGGAT
TAGGGCTGCTTTGGAAGAGAAACTGAGGGATAGCGAGGCCAGAGCTGCCGAACTGACCACCAAGCTGG
AGGCCACTGTGGCCGCCAAATCAAGTGCCGAGCAAGAGAGAGAGAACATCAAAGTGGCAGTCGAGGCC
ACTGAGCTGGAGAGAGCCCAGGAAGAGGCTGAAAGGCTGGCAGGCGACCTGGAGAAAGCTGAGGAGGA
GGCAGAAAGACTGGCAGGCGACCTGGAAAAAGCCCAAGAGGAAGCTGAGACGCTGGCTGGCGTCAACG
AGCTGGCTGACAAGGACCCAGAATTGGCCGCCTTTAGGGAAAAGCGCAGGGCCGCTCACGGAGCCAGA
GCAGACGAACCCGAGCTGGCTGCTGCCGACGGGATTAGCACACGCAATGCCAGGGCCGGAAGCCGTGG
ACGTCCAGCCGCACAGATCAATCCCGCTGCTGAAGCCGTGGATCCCGTGACTATCGCAGCTGAGCCAC
TGTACGCCGTGACCCTCGACGAATACAAGGCCAAACAGACCGCACTGGAAAACGCAGTTGAAGTGGCC
TGCGCAGCCGAAGAGACTGTGAAAGAGAAACTGAGGGAGAACAGCGACCTGATGGTGGAGCTGGAAAA
GGTGCGTGACCAGGCTTACGAGATGGATAGGAGGAGGCAAGAAGACGGAGCCGCAATGGAAGGGGAGC
TGCTGGTTGTGCTGATGGAGCTCAAGAAACTCAAGGGAATCAACGACGCCCTGCTGGCTGTGCTTAGG
GACAAAGAGTGTGAGGTGAAAGAGCTTCGATACCACAACGAGTTGTGGGTTGACCCAACGGGAGACAA
GAAGCAGGTGGTGACGAGGCACACTAAGATCTTTGACGGCAACTGGGAGAGGATTGTGCGAGAACGAC
CCGAAGGGCTGTTCGCAGCCTTTGTGATCGATAGCAGTAACGCCTGCCACGTCCCTGGGGACAACATC
AAACAGGTGTCTTTTGACCACGACGTCGACATGGCACTGCAGAGGGTTCGTGAAGCAGCAGAAAAAGC
GAAATGCGAATTAAGCAGTGCGATGGAAACAGAAGTTAATTTACCATTTATTACCGCTAACGCAGATG
GCGCGCAACATATCCAGATGCGCATTTCTCGTTCTAAATTCGAAGGTATCACCCAGAGGTTGATTGAT
CGTTCAATCGCCCCGTGTAAACAGTGTATGAAGGATGCAGGGGTTGAGCTCAAAGAAATTAATGATGT
TGTGCTTGTTGGCGGGATGACAAGAATGCCAAAAGTTGTTGAAGAAGTAAAAAAGTTTTTTCAAAAAG
ATCCCTTTCGCGGCGTGAATCCCGACGAGGCCGTCGCTCTTGGTGCGGCCACCCTGGGCGGAGTTCTG
CGTGGTGATGTCAAGGGTTTAGTGTTGCTGGATGTGACACCTTTGTCACTGGGAATTGAGACTCTGGG
TGGTGTCTTTACTCGTATGATACCGAAAAACACTACCATTCCCACGAAAAAGTCGCAGACCTTTTCTA
CTGCTGCCGACAATCAGACACAGGTCGGAATTAAGGTTTTTCAAGGTGAGCGTGAAATGGCTGCTGAC
AACCAGATGATGGGGCAGTTCGACCTGGTTGGGATTCCGCCCGCACCTAGGGGGGTGCCCCAAATCGA
AGTTACCTTCGACATAGACGCCAATGGTATCTGTCATGTCACAGCAAAAGATAAAGCAACGGGTAAAA
CACAGAATATTACGATCACAGCAAACGGAGGATTGAGTAAAGAGCAGATTGAACAGATGATTCGCGAT
TCGGAACAACATGCTGAGGCCGATCGTGTCAAGCGTGAACTGGTCGAGGTTAGAAATAATGCGGAGAC
CCAGCTTACCACCGCGGAACGTCAATTAGGTGAATGGAAGTATGTTTCCGATGCAGAAAAGGAGAATG
TAAAAACGCTGGTCGCCGAGTTGCGTAAGGCAATGGAAAATCCAAATGTTGCGAAAGACGATCTGGCT
GCAGCAACAGACAAACTACAAAAGGCCGTAATGGAATGCGGCAGAACCGAGTATCAGCAAGCCGCCGC
TGCTAACAGCGGCTCTACGTCGAATAGCGGTGAACAACAGCAGCAACAAGGCCAGGGAGAACAACAGC
AACAGCAGTCTCAGGGAGAGGAAACAAAACTCGAGCATCATCATCATCATCATTGAAAGCTT
