# Supplementary material for: Gene design, optimization of protein expression and preliminary evaluation of a new chimeric protein for the serological diagnosis of both human and canine visceral leishmaniasis
Source: PLoS Negl Trop Dis. 2020 Jul 27;14(7):e0008488. doi: 10.1371/journal.pntd.0008488 (PMC7410341; doi:10.1371/journal.pntd.0008488)
Supplement: S12 Fig — The sequence also shows the pSS-gIII peptide, in pink, and the T7 tag epitope, in light blue. Fragments corresponding to the regions encoding the repeats from Lci12, Lci2 and Lci3 are in green, orange and dark blue, respectively, and elements introduced during the synthesis and cloning procedures in purple. The C-terminal His-Tag is in red. The Lci3 fragment lacking repeats is in brown. (PDF) [file pntd.0008488.s013.pdf]

Supporting Figure S12. Full length amino acid sequence of the recombinant Q1SX protein. The sequence also shows the pSS-gIII peptide, in pink, and the T7 tag epitope, in light blue. Fragments corresponding to the regions encoding the repeats from Lci12, Lci2 and Lci3 are in green, orange and dark blue, respectively, and elements introduced during the synthesis and cloning procedures in purple. The C-terminal His-Tag is in red. The Lci3 fragment lacking repeats is in brown.

MAKKLLFAIPLVVPFYSHTMASMTGGQQMGRMIEAEEQARREAEEQARRVAEEQARREAEEQAR
REVELEEKLRGTEARAAELAARLKAIAAMKASMVQERESARDALEEKLRGSEVRAAELAARLKA
AVAAKSSAEQDRENTRATLEQRLRESEERAAELASQLEAAAAAKSSAEQDRENTRAALEEKLRG
SEERAAELGTRVKASSAAKALAEQERDRIRAALEEKLRDSEARAAELTTKLEATVAAKSSAEQE
RENIKVAVEATELERAQEEAERLAGDLEKAEEEAERLAGDLEKAQEEAETLAGVNELADKDPEL
AAFREKRRAAHGARADEPELAAADGISTRNARAGSRGRPAAQINPAAEAVDPVTIAAEPLYAVT
LDEYKAKQTALENAVEVACAAEETVKEKLRENSDLMVELEKVRDQAYEMDRRRQEDGAAMEGEL
LVVLMELKKLKGINDALLAVLRDKECEVKELRYHNELWVDPTGDKKQVVTRHTKIFDGNWERIV
RERPEGLFAAFVIDSSNACHVPGDNIKQVSFDHDHHHHHH
