# Supplementary material for: Gene design, optimization of protein expression and preliminary evaluation of a new chimeric protein for the serological diagnosis of both human and canine visceral leishmaniasis
Source: PLoS Negl Trop Dis. 2020 Jul 27;14(7):e0008488. doi: 10.1371/journal.pntd.0008488 (PMC7410341; doi:10.1371/journal.pntd.0008488)
Supplement: S13 Fig — The repeats found in D2, but missing from Q1SX, are boxed (PDF) [file pntd.0008488.s014.pdf]

Supporting Fig. S13. Alignment comparing the amino acid sequences of the D2 and Q1SX chimeras, highlighting the Lci3 repeats missing from Q1SX.

The repeats found in D2, but missing from Q1SX are boxed

```
Q1SX MAKKLLFAIP LVVPFYSHTM ASMTGGQQMG R--------- ----MDEAEE QARREAEEQA RRVAEEQARR EAEEQARREV 67
D2           MR GSHHHHHHGM ASMTGGQQMG RDLYDDDDKD RWGSELEAEE QARREAEEQA RRVAEEQARR EAEEQARREV 72

Q1SX ELEEKLRGTE ARAAELAARL KAIAAMKASM VQERESARDA LEEKLRGSEV RAAELAARLK AAVAAKSSAE QDRENTRATL 147
D2   ELEEKLRGTE ARAAELAARL KAIAAMKASM VQERESARDA LEEKLRGSEV RAAELAARLK AAVAAKSSAE QDRENTRATL 152

Q1SX EQRLRESEER AAELASQLEA AAAAKSSAEQ DRENTRAALE EKLRGSEERA AELGTRVKAS SAAKALAEQE RDRIRAALEE 227
D2   EQRLRESEER AAELASQLEA AAAAKSSAEQ DRENTRAALE EKLRGSEERA AELGTRVKAS SAAKALAEQE RDRIRAALEE 232

Q1SX KLRDSEARAA ELTTKLEATV AAKSSAEQER ENIKVAVE-- ---------- ---------- ---------- ---------- 265
D2   KLRDSEARAA ELTTKLEATV AAKSSAEQER ENIKVAVEEL QKAQEDGERQ KADNRQLASD NERLATELER AQEEAERLAG 312

Q1SX ---------- ---------- ---------- ---------- ---------- ----ATELER AQEEAERLAG DLEKAEEEAE 291
D2   DLEKAEEEAE RLAGDLEKAQ EEAETLAGEL QKAQEDGERQ KADNRQLASD NERLATELER AQEEAERLAG DLEKAEEEAE 392

Q1SX RLAGDLEKAQ EEAETLAGVN ELADKDPELA AFREKRRAAH GARADEPELA AADGISTRNA RAGSRGRPAA QINPAAEAVD 371
D2   RLAGDLEKAQ EEAETLAGVD ELADKDPELA AFREKRRAAH GARADEPELA AADGISTRNA RAGSRGRPAA QINPAAEAVD 472

Q1SX PVTIAAEPLY AVTLDEYKAK QTALENAVEV ACAAEETVKE KLRENSDLMV ELEKVRDQAY EMDRRRQEDG AAMEGELLVV 451
D2   PVTIAAEPLY AVTLDEYKAK QTALENAVEV ACAAEETVKE KLRENSDLMV ELEKVRDQAY EMDRRRQEDG AAMEGELLVV 552

Q1SX LMELKKLKGI NDALLAVLRD KECEVKELRY HNELWVDPTG DKKQVVTRHT KIFDGNWERI VRERPEGLFA AFVIDSSNAC 531
D2   LMELKKLKGI NDALLAVLRD KECEVKELRY HNELWVDPTG DKKQVVTRHT KIFDGNWERI VRERPEGLFA AFVIDSSNAC 632

Q1SX HVPGDNIKQV SFDHDHHHHH H 552
D2   HVPGDNIKQV SFDHDEFEA    651
```
