# Supplementary material for: Gene design, optimization of protein expression and preliminary evaluation of a new chimeric protein for the serological diagnosis of both human and canine visceral leishmaniasis
Source: PLoS Negl Trop Dis. 2020 Jul 27;14(7):e0008488. doi: 10.1371/journal.pntd.0008488 (PMC7410341; doi:10.1371/journal.pntd.0008488)
Supplement: S14 Fig — The sequence also shows the flanking XbaI and HindIII sites, as well as the internal SalI and XhoI sites flanking the segment encoding the extra Lci3 repeats, in gray (all restriction sites are underlined). The segment encoding the pSS-gIII peptide, flanked by the two NcoI sites, is in pink, while the segment encoding the T7 tag epitope is in light blue. Segments encoding the repeats from Lci12, Lci2 and Lci3 also found in the Q1 constructs are in green, orange and dark blue, respectively, while the fragment encoding the His-Tag is in red. (PDF) [file pntd.0008488.s015.pdf]

Supporting Figure S14. Full length nucleotide sequence of the recombinant Q5 gene. The sequence also shows the flanking XbaI and HindIII sites, as well as the internal SalI and XhoI sites flanking the extra Lci3 repeats segment, in grey (all restriction sites are underlined). The segment encoding the pSS-gIII peptide, flanked by the two NcoI sites, is in pink, while the segment encoding the T7 tag epitope is in light blue. Segments encoding the repeats from Lci12, Lci2 and Lci3 also found in the Q1 constructs are in green, orange and dark blue, respectively, while the fragment encoding the His-Tag is in red.

TCTAGAAGGAGGTAAACCATGGCAAAAAAACTGCTGTTCGCGATTCCGCTGGTGGTGCCGTTCT
ATAGCCATACCATGGCTAGCATGACTGGTGGACAGCAAATGGGTCGGATGATCGAGGCCGAGGA
ACAGGCCAGGAGGGAGGCTGAAGAGCAGGCCAGACGCGTCGCCGAGGAACAGGCCAGGAGGGAG
GCAGAGGAGCAAGCCAGGAGAGAGGTCGAGCTTGAAGAGAAACTGAGGGGAACTGAAGCCAGAG
CTGCCGAACTCGCCGCCAGGCTGAAGGCCATTGCTGCCATGAAAGCAAGCATGGTGCAGGAAAG
GGAGTCCGCACGCGACGCACTGGAAGAAAAGCTGAGGGGCAGCGAGGTGAGGGCCGCAGAGCTC
GCAGCCAGACTCAAAGCCGCAGTGGCAGCCAAAAGCAGCGCAGAACAGGATAGAGAAAACACGA
GAGCCACCCTGGAACAGAGACTGAGGGAGAGTGAGGAAAGGGCCGCAGAGCTGGCCAGTCAGCT
GGAAGCAGCCGCAGCCGCAAAGAGCAGCGCAGAGCAGGACAGGGAAAACACACGAGCAGCCCTG
GAGGAAAAGCTGAGGGGATCAGAGGAGAGGGCTGCAGAGCTGGGCACCCGAGTCAAGGCCAGCA
GCGCCGCAAAGGCCCTTGCCGAGCAGGAACGCGATAGGATTAGGGCTGCTTTGGAAGAGAAACT
GAGGGATAGCGAGGCCAGAGCTGCCGAACTGACCACCAAGCTGGAGGCCACTGTGGCCGCCAAA
TCAAGTGCCGAGCAAGAGAGAGAGAACATCAAAGTGGCAGTCGAGGTCGACGAGCTGCAGAAAG
CCCAGGAGGACGGCGAACGTCAAAAGGCAGACAATAGACAGCTGGCCTCAGACAACGAGAGACT
GGCCACCGAGCTGGAAAGAGCTCAGGAGGAAGCAGAGCGCCTGGCCGGAGACCTGGAGAAAGCA
GAAGAAGAGGCTGAGCGACTTGCAGGCGACCTGGAGAAGGCCCAGGAAGAGGCAGAAACACTGG
CTGGGGAGCTCCAAAAGGCCCAGGAGGACGGGGAACGTCAAAAGGCAGACAATCGGCAGCTGGC
CTCAGACAACGAAAGGCTGCTCGAGGCCACTGAGCTGGAGAGAGCCCAGGAAGAGGCTGAAAGG
CTGGCAGGCGACCTGGAGAAAGCTGAGGAGGAGGCAGAAAGACTGGCAGGCGACCTGGAAAAAG
CCCAAGAGGAAGCTGAGACGCTGGCTGGCGTGGACGAGCTGGCTGACAAGGACCCAGAATTGGC
CGCCTTTAGGGAAAAGCGCAGGGCCGCTCACGGAGCCAGAGCAGACGAACCCGAGCTGGCTGCT
GCCGACGGGATTAGCACACGCAATGCCAGGGCCGGAAGCCGTGGACGTCCAGCCGCACAGATCA
ATCCCGCTGCTGAAGCCGTGGATCCCGTGACTATCGCAGCTGAGCCACTGTACGCCGTGACCCT
CGACGAATACAAGGCCAAACAGACCGCACTGGAAAACGCAGTTGAAGTGGCCTGCGCAGCCGAA
GAGACTGTGAAAGAGAAACTGAGGGAGAACAGCGACCTGATGGTGGAGCTGGAAAAGGTGCGTG
ACCAGGCTTACGAGATGGATAGGAGGAGGCAAGAAGACGGAGCCGCGATGGAAGGGGAGCTGCT
GGTTGTGCTGATGGAGCTCAAGAAACTCAAGGGAATCAACGACGCCCTGCTGGCTGTGCTTAGG
GACAAAGAGTGTGAGGTGAAAGAGCTTCGATACCACAACGAGTTGTGGGTTGACCCAACGGGAG
ACAAGAAGCAGGTGGTGACGAGGCACACTAAGATCTTTGACGGCAATTGGGAGAGGATTGTGCG
AGAACGACCCGAAGGGCTGTTCGCAGCCTTTGTGATCGATAGCAGTAACGCCTGCCACGTCCCT
GGGGACAACATCAAACAGGTGTCTTTTGACCACGACCATCATCATCATCATCATTGATAAAAGC
TT
