# Supplementary material for: Gene design, optimization of protein expression and preliminary evaluation of a new chimeric protein for the serological diagnosis of both human and canine visceral leishmaniasis
Source: PLoS Negl Trop Dis. 2020 Jul 27;14(7):e0008488. doi: 10.1371/journal.pntd.0008488 (PMC7410341; doi:10.1371/journal.pntd.0008488)
Supplement: S15 Fig — The sequence also shows the pSS-gIII peptide, in pink, and the T7 tag epitope, in light blue. Fragments corresponding to the regions encoding the repeats from Lci12 and Lci2 are in green, orange, respectively. The original Lci3 repeats segment found in the Q1 constructs is in dark blue, while the extra Lci3 repeats segment is in grey and its non-repeats region in brown. Elements introduced during the synthesis and cloning procedures are in purple. The C-terminal His-Tag is in red. (PDF) [file pntd.0008488.s016.pdf]

Supporting Figure S15. Full length amino acid sequence of the recombinant Q5 protein. The sequence also shows the pSS-gIII peptide, in pink, and the T7 tag epitope, in light blue. Fragments corresponding to the regions encoding the repeats from Lci12 and Lci2 are in green, orange, respectively. The original Lci3 repeats segment found in the Q1 constructs is in dark blue, while the extra Lci3 repeats segment is in grey and its non-repeats region in brown. Elements introduced during the synthesis and cloning procedures are in purple. The C-terminal His-Tag is in red.

MAKKLLFAIPLVVPFYSHTMASMTGGQQMGRMIEAEEQARREAEEQARRVAEEQARREAEEQARREVELE
EKLRGTEARAAELAARLKAIAAMKASMVQERESARDALEEKLRGSEVRAAELAARLKAAVAAKSSAEQDR
ENTRATLEQRLRESEERAAELASQLEAAAAAKSSAEQDRENTRAALEEKLRGSEERAAELGTRVKASSAA
KALAEQERDRIRAALEEKLRDSEARAAELTTKLEATVAAKSSAEQERENIKVAVEVDELQKAQEDGERQK
ADNRQLASDNERLATELERAQEEAERLAGDLEKAEEEAERLAGDLEKAQEEAETLAGELQKAQEDGERQK
ADNRQLASDNERLLEATELERAQEEAERLAGDLEKAEEEAERLAGDLEKAQEEAETLAGVDELADKDPEL
AAFREKRRAAHGARADEPELAAADGISTRNARAGSRGRPAAQINPAAEAVDPVTIAAEPLYAVTLDEYKA
KQTALENAVEVACAAEETVKEKLRENSDLMVELEKVRDQAYEMDRRRQEDGAAMEGELLVVLMELKKLKG
INDALLAVLRDKECEVKELRYHNELWVDPTGDKKQVVTRHTKIFDGNWERIVRERPEGLFAAFVIDSSNA
CHVPGDNIKQVSFDHDHHHHHH
